# Region-specific human cardiac extracellular vesicles differentially regulate mitochondrial function

**DOI:** 10.20517/evcna.2026.58

**Published:** 2026-09-28

**Authors:** Genevieve C. Sparagna, Christina M. Coughlan, Hailey L. Chapman, Sydney R. Shuff, Ashley E. Pietra, Benjamin J. Kopecky, Alicia J. Chapman, Eva M. Medina, Anthony J. Saviola, Kathryn C. Chatfield, Shelley D. Miyamoto, Anastacia M. Garcia

**Affiliations:** ^1^Department of Medicine, Division of Cardiology, University of Colorado Anschutz Medical Campus, Aurora, CO 80045, USA.; ^2^Department of Neurology, University of Colorado Anschutz, Aurora, CO 80045, USA.; ^3^Department of Life Sciences, Division of Systems and Synthetic Biology, Chalmers University of Technology, Gothenburg 41296, Sweden.; ^4^Department of Pediatrics, Division of Cardiology, University of Colorado Anschutz Medical Campus and Children’s Hospital Colorado, Aurora, CO 80045, USA.; ^5^Department of Biochemistry and Molecular Genetics, University of Colorado Anschutz Medical Campus, Aurora, CO 80045, USA.; ^#^These authors are joint first authors.; ^†^These authors are joint senior authors.

**Keywords:** Cardiolipin, phospholipids, interfibrillar, mitochondria, subsarcolemmal, proteomics, seahorse, liquid chromatography mass spectrometry

## Abstract

**Aim:** The human heart contains two major mitochondrial subtypes, *subsarcolemmal (SSL)*, fueling surface ion channels, and *interfibrillar (IF)*, fueling interior contractile machinery. This study investigated whether the distinct extracellular vesicles (EVs) that we determined to be associated with these regions could influence cardiac energetics.

**Methods:** Following mitochondrial extraction, Subsarcolemmal Heart Extracellular Vesicles (SHEVs) and Interfibrillar Heart Extracellular Vesicles (IHEVs) were extracted from nonfailing human left ventricular lysates using precipitation-based methods. We characterized these EVs by size, proteomic profile, phospholipid content, and their ability to modulate mitochondrial function in primary neonatal rat ventricular myocytes (NRVMs).

**Results:** SHEVs (mean size: 148.9 ± 25.9 nm) and IHEVs (102.9 ± 31.0 nm) exhibited distinct proteomic signatures. SHEV and IHEV contained mostly structural proteins, but also contained 8% and 4% mitochondrial and glycolytic proteins, respectively. Lipidomic analysis revealed higher sphingomyelin and lower cardiolipin in EVs compared to whole tissue. SHEV treatment significantly increased mitochondrial ATP production, basal, and maximal respiration in NRVMs (*P* < 0.05). Conversely, IHEVs decreased maximal respiration without altering ATP production. Neither EV type exhibited intrinsic mitochondrial activity.

**Conclusion:** Regionally distinct EVs within the human heart have the capacity to modulate mitochondrial function. These findings suggest that cardiac EVs may act as localized regulators of cardiomyocyte bioenergetics.

## INTRODUCTION

Reflecting its exceptional energetic demands, the heart contains the highest mitochondrial density of any organ. These demands arise from the need to power both electrical excitation through ion channel activity and the contractile machinery within cardiomyocytes, composed of sarcomeric proteins that include actin and myosin. The mitochondria required to sustain these processes, subsarcolemmal (SSL) for the ion channels and interfibrillar (IF) for the sarcomeric fibers, are located next to, and often intertwined with, the specific proteins that require ATP^[1]^. While it is unusual for a tissue to have multiple mitochondrial types, the two major cardiac mitochondrial subtypes are among the best characterized of any tissue. The method most widely used for separating SSL and IF mitochondria from heart tissue was first published by the Hoppel lab in 1977^[2]^. This isolation process involves homogenization and differential centrifugation to remove SSL mitochondria from the outer region of the cardiomyocyte, followed by gentle enzymatic digestion of cytoskeletal proteins to release the IF mitochondria, and finally high-speed differential centrifugation to isolate the IF mitochondria. These two mitochondrial subtypes differ not only in location but also in shape, ultrastructure, and oxygen consumption rate, with IF mitochondria having a higher respiration rate^[2,3]^. Our laboratory modified the original isolation method developed in rat heart to isolate SSL and IF mitochondria from fresh human heart tissue, and, to our knowledge, this is the first study to isolate extracellular vesicles (EVs) associated with both cardiac mitochondrial subpopulations from human heart.

Mitochondrial-derived vesicles (MDVs) are formed in the heart under normal and pathological conditions and are considered part of the first-line response to stress^[4]^. Mild mitochondrial damage has been shown to increase the release of EVs that contain unpackaged damaged mitochondrial components in an attempt to protect heart cells from this toxic material and prevent the formation of an inflammatory *milieu*^[5]^. Several groups report that MDVs alter heart mitochondria during disease states^[6]^. This has expanded interest in MDVs beyond understanding their molecular cargo to determining their role in heart energetics after secretion. MDVs are highly heterogeneous in size and function, with this profile being cell-type dependent^[7]^. In human cardiac tissue, it remains unclear from which mitochondrial subpopulations these vesicles arise, and whether the vesicles enriched and identified in our study can be characterized as MDVs based on their size, cargo, and function.

In this study, vesicles were isolated after extraction of SSL and IF mitochondria from fresh nonfailing human left ventricular cardiac tissue obtained from donor hearts. The SHEV and IHEV EVs were extracted from respective supernatants, and their physical and functional properties were investigated. Despite the small sample size in this pilot study, the findings were highly consistent across the EV populations isolated from biologically distinct donors.

## METHODS

### Chemicals and reagents

Unless otherwise noted, all chemicals were from Sigma (St. Louis, MO, US). BIOPS biopreservation solution was made using the Veksler *et al*. formulation^[8]^.

### Patient samples

Donor hearts included in this study were obtained in 2021 through the Institutional Review Board-approved University of Colorado Adult Cardiac Tissue Bank, approval number COMIRB 01-568. Explanted left ventricular tissue from nonfailing donor hearts from adults of all races, genders, and ethnic backgrounds was included, and all participants provided informed consent. Nonfailing left ventricular tissue was procured from donors with normal systolic ventricular function (ejection fraction > 50%) whose hearts were not used for transplant due to lack of an appropriate recipient. Small pieces of left ventricular heart tissue, about 5 mm per side, were immediately placed in BIOPS preservation solution at 4 °C and maintained in this solution until processing^[8]^. Mitochondria were isolated from fresh tissue within 12 h of donor-heart excision, and supernatant samples were frozen at -80 °C until processing for EVs occurred once all donor hearts were collected.

### Mitochondrial isolation

Subpopulations of myocardial mitochondria, SSL, located beneath the plasma membrane, and IF, residing between the myofibrils, were isolated using differential centrifugation and trypsin digestion as previously described by Palmer *et al.* with some modifications for human tissue^[2]^. Briefly, freshly explanted cardiac tissue pieces, no larger than 5 mm, were placed in ice-cold BIOPS solution. Tissue was trimmed of fat, placed into CP1 mitochondrial isolation buffer as described in Palmer *et al*., and minced using scissors^[2]^. Low-speed centrifugations were performed at 1,500 × *g* and high-speed at 4,200 × *g*. One mg of trypsin was used per gram of tissue. Mitochondria were resuspended in potassium buffer, and protein was quantified by bicinchoninic acid (BCA) assay (Thermo Scientific Pierce, Waltham, MA, US).

### Isolation of vesicles

Figure 1 shows a schematic of isolating the two major types of heart mitochondria, SSL and IF, and extracting the two associated vesicle types, SHEV and IHEV, respectively. After high-speed centrifugation (4,200 × *g*) supernatants were collected and pooled and then frozen until further processing. The collected supernatants were then thawed, and precipitation was performed using a ratio of 67 µL ExoQuick (cat # EXOQ-20A1) per 250 µL of supernatant. This solution was incubated on ice for 30 min, then centrifuged at 4 °C for 10 min at 1,000 × *g*. The EV pellets were resuspended in the same volume of PBS as the starting supernatant and stored at -80 °C until use.

**Figure 1:**
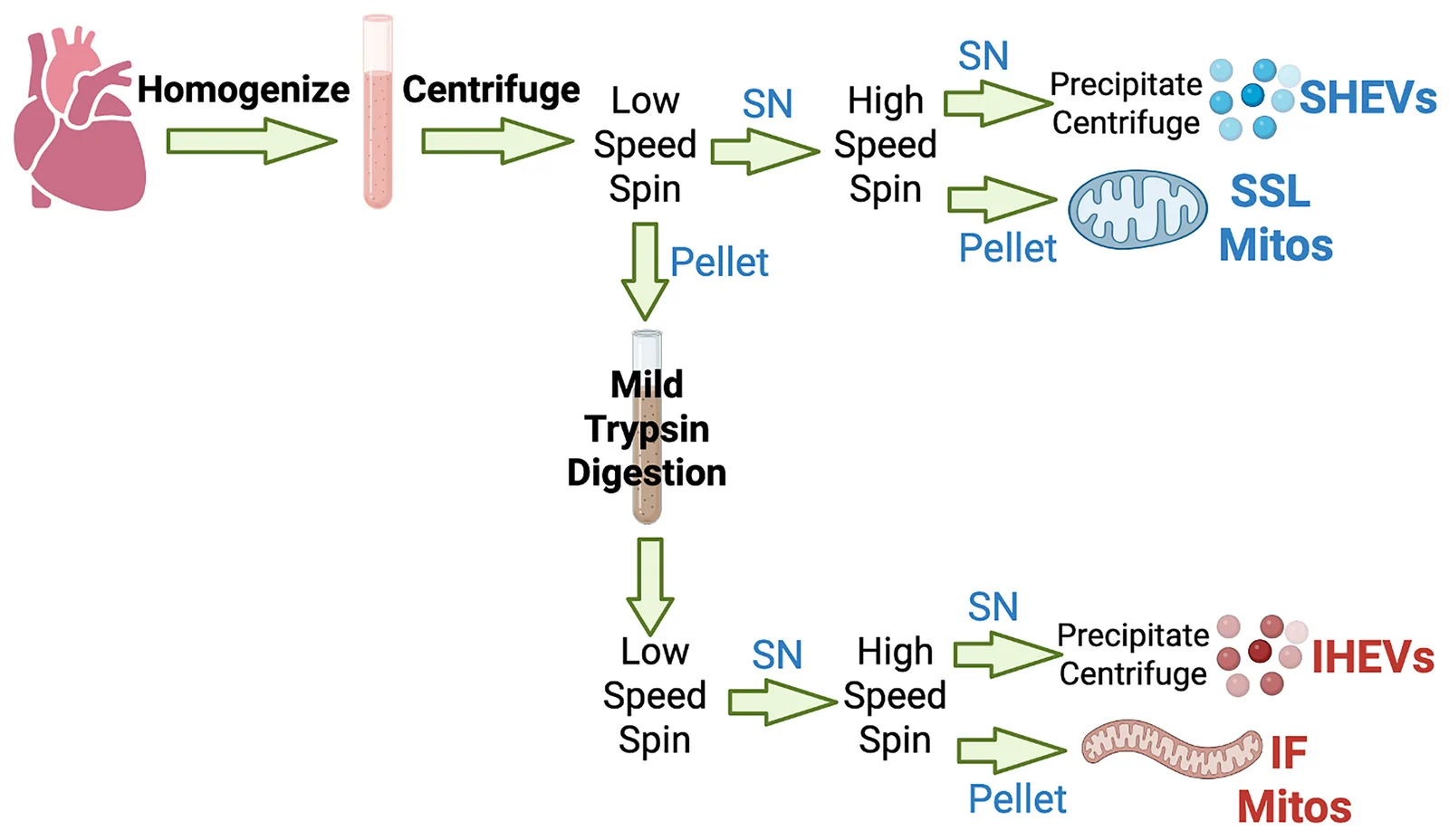
Diagram of the isolation method of SSL and IF mitochondria, and SSL- and IF-associated heart vesicles (SHEVs and IHEVs, respectively). Created in BioRender. Garcia, A. (2026) https://BioRender.com/y27z9wp. SSL: Subsarcolemmal; IF: interfibrillar; SHEV: subsarcolemmal heart extracellular vesicle; IHEV: interfibrillar heart extracellular vesicle; SN: supernatant.

### Quantification of EVs with nanosight

Total EVs isolated from SHEV and IHEV samples were assessed for their sizes and concentrations using the Nanosight nanoparticle tracking analysis (NTA) instrument (NS300, Software Version: NTA 3.2 Dev Build 3.2.16; Malvern Panalytical, Malvern WR14 1XZ, UK). For each measurement, the EVs were freshly diluted 1:100 in ddH_2_O before reading, and the reported EV concentration was adjusted accordingly. Average curves for SHEVs and IHEVs are shown in Supplementary Figures 1 and 2, respectively.

### Assessment of canonical EV tetraspanin markers with leprechaun

To determine the presence of EV-associated tetraspanins in the SHEV and IHEV preparations, Leprechaun analysis was performed. EVs were analyzed using Tetraspanin kits (Unchained Labs: 251-1044) according to the manufacturer’s instructions. In summary, these chips capture small EVs (sEVs) based on the three most commonly reported tetraspanins (CD63, CD81, and CD9) and also contain murine IgG spots which serve as controls. The captured EVs can subsequently be co-stained with fluorescently conjugated antibodies (custom or, in this work, tetraspanins, the latter being provided in kit 251-1044) to assess the number and size of the sEVs of each subtype (CD63, CD81, and CD9) that also contain the other tetraspanins (e.g., how many CD9-captured sEVs also have CD63 and CD81). For each co-staining antibody, fluorescently tagged CD63-647, CD9-488, and CD81-555 were utilized for staining.

### Mass spectrometry-based proteomics

EVs were prepared for mass spectrometry using S-Trap^TM^ microfilters (Protifi, Huntington, NY) according to the manufacturer’s protocol. Digested peptides were cleaned using Pierce^TM^ C18 Spin Tips (Thermo Scientific) according to the manufacturer’s protocol, dried in a vacuum centrifuge, and resuspended in 0.1% formic acid in mass spectrometry-grade water.

Preparation of heart samples was performed as previously described^[9]^. Briefly, lyophilized samples were processed by stepwise extraction with CHAPS and high-salt guanidine hydrochloride, followed by chemical digestion with hydroxylamine hydrochloride in guanidine hydrochloride, generating cellular, soluble extracellular matrix (ECM), and insoluble ECM fractions for each sample, respectively. The protein concentration of each fraction for each sample was measured using the A660 Protein Assay (Pierce^TM^). 30 mg of protein resulting from each fraction was subjected to proteolytic digestion using a filter-aided sample preparation protocol with 10 kDa molecular weight cutoff filters (Sartorius Vivacon 500 #VN01H02). Samples were reduced with 5 mM tris(2-carboxyethyl)phosphine, alkylated with 50 mM 2-chloroacetamide, and digested overnight with trypsin (enzyme-to-substrate ratio of 1:100) at 37 °C. Peptides were recovered from the filter using successive washes with 0.2% formic acid.

Digested peptides were loaded onto individual Evotips following the manufacturer’s protocol and separated on an Evosep One chromatography system (Evosep, Odense, Denmark) using a Pepsep column (150 μm inner diameter, 15 cm) packed with ReproSil C18 1.9 μm, 120 Å resin. The system was coupled to a timsTOF Pro mass spectrometer (Bruker Daltonics, Bremen, Germany) via the nano-electrospray ion source (Captive Spray, Bruker Daltonics). The mass spectrometer was operated in Parallel Accumulation-Serial Fragmentation (PASEF) mode. We set the ramp time to 100 ms and acquired 10 PASEF tandem mass spectrometry (MS/MS) scans per acquisition cycle. MS and MS/MS spectra were recorded from m/z 100 to 1,700. The ion mobility was scanned from 0.7 to 1.50 Vs/cm^2^. Precursors for data-dependent acquisition were isolated within ±1 mass-to-charge (m/z) unit and fragmented with an ion mobility-dependent collision energy, which increased linearly from 20 to 59 eV in positive mode. Low-abundance precursor ions with an intensity above a threshold of 500 counts but below a target value of 20,000 counts were repeatedly scheduled and otherwise dynamically excluded for 0.4 min.

Fragmentation spectra were searched against the UniProt human proteome database using the MSFragger-based FragPipe computational platform^[10]^. Contaminants and reverse decoys were automatically added to the database*.* The precursor-ion mass tolerance and fragment-ion mass tolerance were set to 15 and 20 ppm, respectively. EVs were searched with fixed modifications set to carbamidomethylation, and variable modifications set to oxidation. Tissue samples were searched with fixed modifications set as carbamidomethyl, and variable modifications were set as oxidation, hydroxyproline oxidation, Gln->pyro-Glu (N-terminus), deamidation, and acetyl (Protein N-terminus). Two missed tryptic cleavages were allowed, and the protein-level false discovery rate was ≤ 1%.

### Classification of proteins

The 3,758 proteins in the proteomics output were categorized using Claude Science, beta version, and spot-checked manually. After normalizing proteins so each category totaled 100%, Claude first matched the proteins against MitoCarta version 3.0, which identified 757 mitochondrial proteins. It was then instructed to create groupings based on the protein content using UniProt Gene Ontology (GO) cellular-component process keyword rules with a curated override list for cardiac paralog families. Five major categories were used: Mitochondria & Glycolysis, Cytoskeleton & Sarcomere, Vesicle Trafficking, ER Golgi & Secretory, and Other Non-mitochondrial. The Other Non-Mitochondrial category was further investigated for SHEV and IHEV against human protein records in Vesiclepedia 2024: an EVs and extracellular particles repository^[11]^, to determine levels of known EV proteins contained within this category.

### Quantification of phospholipids

Eight phospholipid classes were quantified using liquid chromatography coupled to electrospray ionization mass spectrometry in an API 4000 mass spectrometer (Sciex, Framingham, MA). 50 µg of protein (BCA protein assay, Pierce) from isolated mitochondria, vesicles, or tissue (homogenized in PBS) from two different hearts was extracted according to previously published methods with 100 nmol tetramyristalcardiolipin as an internal standard (Avanti Polar Lipids, Alabaster, AL, US). These samples were analyzed using a full scan, allowing separation of phosphatidic acid (PA), phosphatidylcholine (PC), phosphatidylethanolamine (PE), phosphatidylglycerol (PG), phosphatidylinositol (PI), phosphatidylserine (PS), sphingomyelin (SM), and cardiolipin (CL). The quantification of these phospholipids was performed using standard curves generated using the SPLASH LipidoMIX as a reference standard, along with tetraoleoylcardiolipin as a reference standard for cardiolipin and tetramyristalcardiolipin as an internal standard (all from Avanti Polar Lipids). A curve was generated for each individual phospholipid, which was then used to quantify its amount [Supplementary Figure 3]. The percentage of each phospholipid was calculated by combining the individual phospholipid species and determining the percentage that each contributed to each separate phospholipid. Phospholipid molecular species are listed by their m/z in the Supplementary Table 1. The sum of all phospholipids was generated by combining the individual species.

### Isolation and treatment of rat cardiac cells with vesicles

The University of Colorado Institutional Animal Care and Use Committee approved all animal experiments (Approval 01480), and they were conducted in accordance with National Institutes of Health guidelines. Neonatal rat ventricular myocytes (NRVMs) were isolated from ventricles of 2-day-old Sprague-Dawley rats (Charles River Laboratories, Wilmington, MA, USA) by enzymatic digestion as described previously^[12]^. Briefly, neonatal rats were euthanized using rapid decapitation, and cells were isolated by trypsin digestion of their ventricles. Because NRVMs were freshly isolated for each experiment and maintained for less than 6 days without serial passage, routine mycoplasma testing was not performed. Cells were plated at 50,000 cells/well in 0.1% gelatin-coated (Sigma-Aldrich) 96-well Seahorse plates in growth medium containing MEM-Hanks (Thermo Fisher Scientific, cat. #11575032) and 5% bovine calf serum (Gemini BioProducts, cat. #100-506-500) with a minimum of 5 wells per condition. After 24 h, medium was changed to serum-free MEM-Hanks containing insulin (Sigma-Aldrich, cat. #I1882), transferrin (Sigma-Aldrich, cat. #T1283), bovine serum albumin (Sigma-Aldrich, cat. #A4919-25G), vitamin B12 (Sigma-Aldrich, cat. #V6629), and penicillin G (Sigma-Aldrich, cat. #V7794). All media were buffered with HEPES (Gibco/Thermo Fisher Scientific, cat. #15630080; 20 mM final concentration, pH 7.5). EVs in PBS were added to each well for 48 h before the Seahorse assay. NRVMs were treated with a standardized dose of 5 × 10^8^ particles/well, corresponding to approximately 1 × 10^4^ particles per plated cell. This dosage falls within the range of previously published studies on cardiomyocytes^[13-15]^. Following Seahorse analysis, CyQUANT Direct was used to quantify viable-cell abundance and normalize bioenergetic measurements between wells. Untreated wells received an equivalent volume of PBS only, while some wells had no cells in order to test the intrinsic mitochondrial function of the EVs used in this work. Two independent NRVM isolations were used for the functional studies, with each isolation prepared from ventricles pooled from 53 neonatal rats (106 animals total).

### Seahorse assay

The Agilent Cell Mito Stress Test was used to quantify oxygen consumption rates (OCR) and extracellular acidification rates (ECAR). Briefly, a day before the assay, a calibration cartridge was hydrated with sterile Seahorse calibrant solution at 37 °C in a non-CO_2_ incubator overnight. On the day of the assay, the cells were gently washed and supplied with warmed Seahorse media [Dulbecco’s Modified Eagle Medium (DMEM) plus substrates at final concentrations of 1 mM pyruvate, 2 mM glutamine, and 10 mM glucose, with the pH adjusted to 7.4 using NaOH] and were incubated for 45 min - 1 h at 37 °C in a non-CO_2_ incubator to de-gas. Freshly diluted inhibitors were preloaded into the calibration cartridge in three separate ports to achieve final well concentrations of 1.5 µM oligomycin, 1.0 µM carbonyl cyanide-4-(trifluoromethoxy) phenylhydrazone (FCCP), and 0.9 µM each of rotenone/antimycin A. After the Seahorse assay, we normalized data between wells using CyQUANT^TM^ Direct (Thermo Fisher Scientific, Waltham, MA, USA) to quantify live cells per well. Cells were incubated for an hour in CyQUANT dye and imaged with a fluorescent plate reader with excitation set to 485/20 nm and emission 528/20 nm. All parameters of mitochondrial bioenergetics were calculated using Agilent Seahorse Wave Software 2.0.6.

### Statistical analysis

Prism version 11 (GraphPad Software, La Jolla, California, USA) was used for statistical analysis and data visualization. The donor heart was considered the biological unit for analyses of human-derived samples; repeated measurements or assay wells from the same donor-derived preparation were treated as technical replicates and not as independent biological observations. Normality was assessed using the Shapiro-Wilk test for datasets containing more than two biological observations. Given the limited biological sample sizes, results of normality testing were interpreted cautiously. Normality was not assessed for analyses limited to two donor hearts. Treatment effects were analyzed using an ordinary one-way analysis of variance (ANOVA) with Tukey’s test correction for multiple comparisons, with *P* < 0.05 being significant (comparators for each experiment are listed in the figure legends). Error bars indicate the standard error of the mean (SEM). Due to the limited sample size of the phospholipid analysis (*n* = 2), we interpreted the *P*-value cautiously, ensuring a large fold change and low variability to justify the statistical analysis in such a small sample size. For the functional studies, measurements from approximately five technical Seahorse wells treated with each donor-derived EV preparation were averaged to generate a single donor-level value. Treatment effects were calculated relative to the mean untreated response from the same NRVM preparation (Δ = EV-treated donor mean - untreated mean). Donor-level Δ values were compared with the untreated reference (Δ = 0) using two-sided one-sample *t*-tests. Comparisons between SHEV and IHEV donor-level Δ values were performed using Welch’s unpaired *t*-test because complete matched SHEV/IHEV preparations were not available for all donor hearts. Data analyzed by Claude Science in Supplementary Figure 4 used Benjamini-Hochberg correction with *q* < 0.1 reported, since the experimental group (*n* = 4) was small for this type of analysis.

## RESULTS

### Donor heart demographics

Demographics for the donors of the hearts used in this study are shown in Table 1.

**Table 1:** Donor demographics table

**Sample number**	**Age (years)**	**Sex**	**Ethnicity**	**Medications**	**Cause of death**	**Experiments**
1	31.5	Female	Black or African American	None	Anoxia	P, L, S
2	41.6	Female	White	None	Anoxia	P, L, S
3	61.3	Male	White	Vasopressor digoxin	Intracranial hemorrhage/stroke	P, S
4	55.1	Male	Hispanic/Latino	Vasopressor beta blocker	Head trauma/ blunt injury	P, S (SHEV only)

P: Proteomics; L: lipidomics; S: Seahorse; SHEV: subsarcolemmal heart extracellular vesicle.

### Vesicle characteristics

Table 2 lists the concentration, mean size, and mode of the EV particles from the four hearts. Supplementary Figure 1 shows the size profiles measured by Nanosight for SHEVs, and Figure 2 for IHEVs.

**Figure 2:**
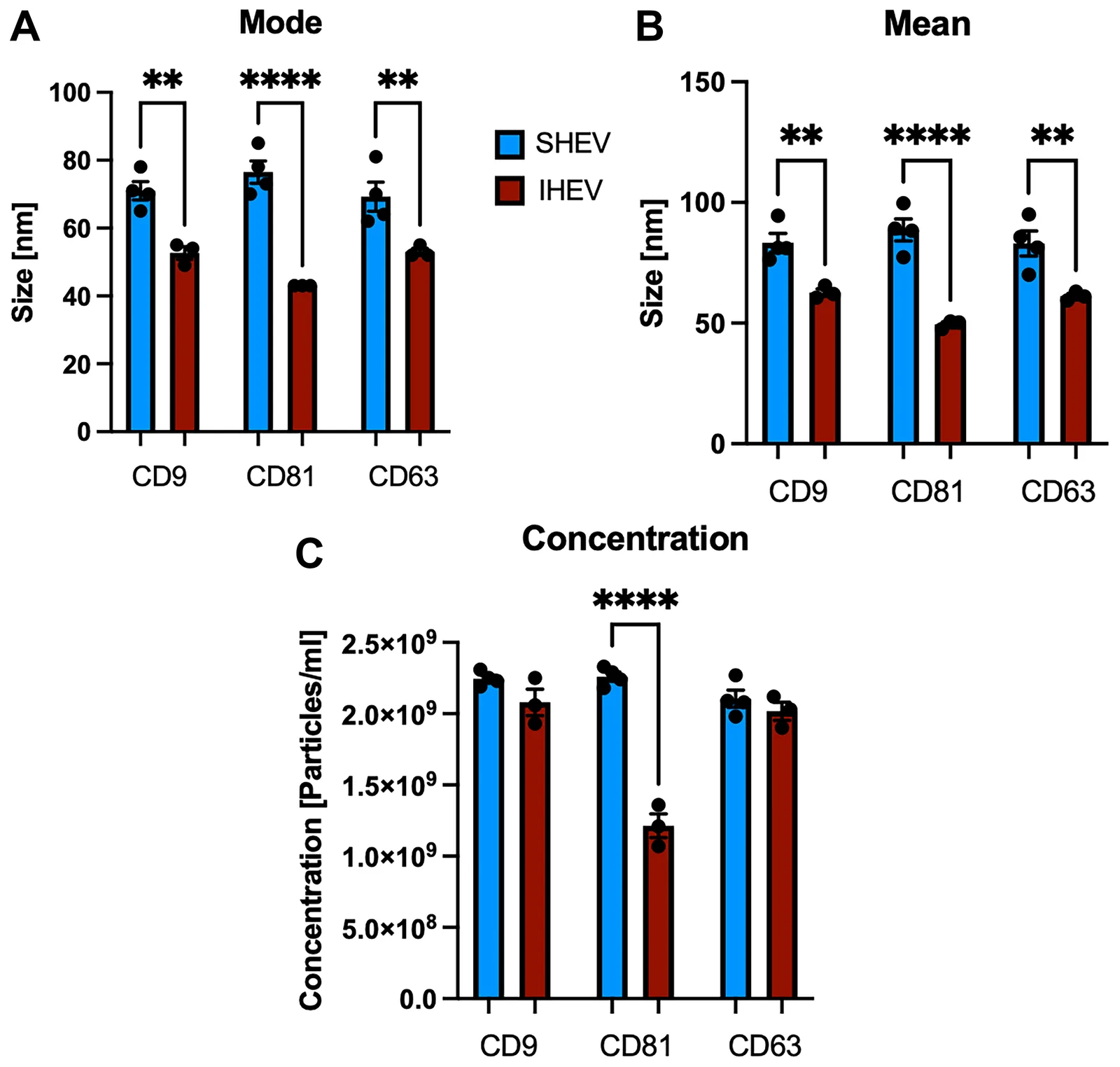
Leprechaun analysis: (A) mode, (B) mean size, and (C) concentration of SHEV and IHEV particles containing CD9, CD81, and CD63. Comparisons were performed only between SHEV and IHEV for each CD protein. ^**^P < 0.01, ^****^P < 0.0001; *N* = 4 SHEV or 3 IHEV donor hearts. Error bars show SEM. Analysis was performed using ordinary one-way ANOVA with multiple comparisons corrected using Tukey’s test. SHEV: Subsarcolemmal heart extracellular vesicle; IHEV: interfibrillar heart extracellular vesicle; SEM: standard error of the mean; ANOVA: analysis of variance.

**Table 2:** Particle sizes from donor hearts

**Sample number**	**Particle type**	**Mean particle size (nm)**	**Mode of particle size (nm)**
1	SHEV	175.1 ± 7.1	83.5 ± 26.0
2	SHEV	166.8 ± 8.1	68.2 ± 21.8
3	SHEV	122.7 ± 10.8	56.6 ± 14.6
4	SHEV	131.1 ± 4.6	56.1 ± 9.4
1	IHEV	120.3 ± 6.6	48.4 ± 14.3
2	IHEV	58.9 ± 2.9	35.3 ± 8.2
3	IHEV	103.9 ± 9.2	49.2 ± 10.1
4	IHEV	128.3 ± 5.4	59.2 ± 8.5

Values are mean ± SD of five separate runs. SHEV: Subsarcolemmal heart extracellular vesicle; IHEV: interfibrillar heart extracellular vesicle; SD: standard deviation.

### Assessment for the presence of EVs as a component of SHEV and IHEV preparations

The Leprechaun (Unchained Labs) and Tetraspanin kits (Unchained Labs: 251-1044) were used to assess the presence of EV-associated tetraspanins based on size and expression. Figure 2 shows the size of the particles containing CD9, CD81, and CD63. Supplementary Figure 5 also shows that, of the seven EVs tested, all four SHEV samples and three IHEV samples (from hearts 1-3) contained CD9, CD63, and CD81. These particle sizes are consistent with their classification as small EVs.

### Proteomics

Mass spectrometry-based proteomics was performed on samples from EVs (SHEV and IHEV) from four separate hearts, and the SSL and IF mitochondria and whole left ventricular (tissue) homogenate from two of these hearts based on limited sample availability. Figure 3 displays an overlap in the amount of protein detected in these samples with mitochondrial proteins identified using the Mitocarta database^[16]^. Because the mitochondrial samples are not highly purified and heart mitochondria are closely associated with endoplasmic reticulum (ER) and structural proteins, their proteomic profiles also contained non-mitochondrial proteins. The SSL and IF mitochondria contained 71.2 ± 2.8 and 82.6 ± 0.3 percent mitochondrial proteins, respectively, whereas whole heart tissue contained 14.8 ± 0.2 percent mitochondrial proteins. The SHEV and IHEV preparations contained even fewer mitochondrial proteins: 5.5 ± 0.9 and 3.8 ± 0.3 percent, respectively. The hierarchical clustering of these five cellular fractions based on protein content for these samples is shown in Figure 4. As expected, the two mitochondrial subtypes cluster together, with SHEV and IHEV more closely related to mitochondria than to whole tissue.

**Figure 3:**
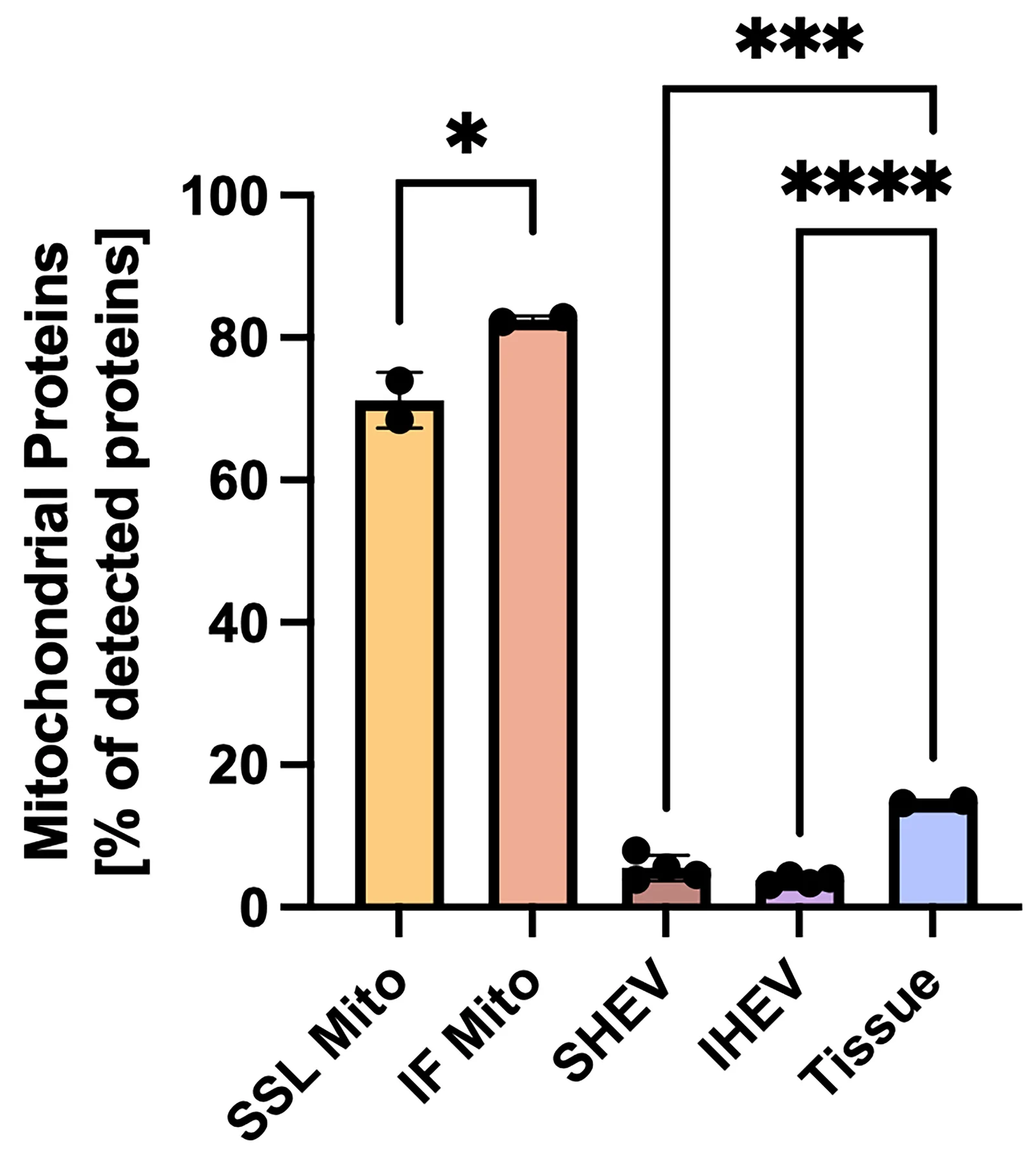
Percentage of detected proteins that are mitochondrial in SSL or IF mitochondria, or SSL- or IF-associated heart EVs (SHEV, IHEV, respectively), or left ventricular tissue. For clarity, statistical comparisons between mitochondria and vesicle or tissue samples are not shown. ^*^P < 0.05, ^***^P < 0.001, ^****^P < 0.0001; *N* = 2-4 donor hearts. Error bars show SEM. Analysis was performed using ordinary one-way ANOVA with multiple comparisons corrected using Tukey’s test. SSL: Subsarcolemmal; IF: interfibrillar; EV: extracellular vesicle; SHEV: subsarcolemmal heart extracellular vesicle; IHEV: interfibrillar heart extracellular vesicle; SEM: standard error of the mean; ANOVA: analysis of variance.

**Figure 4:**
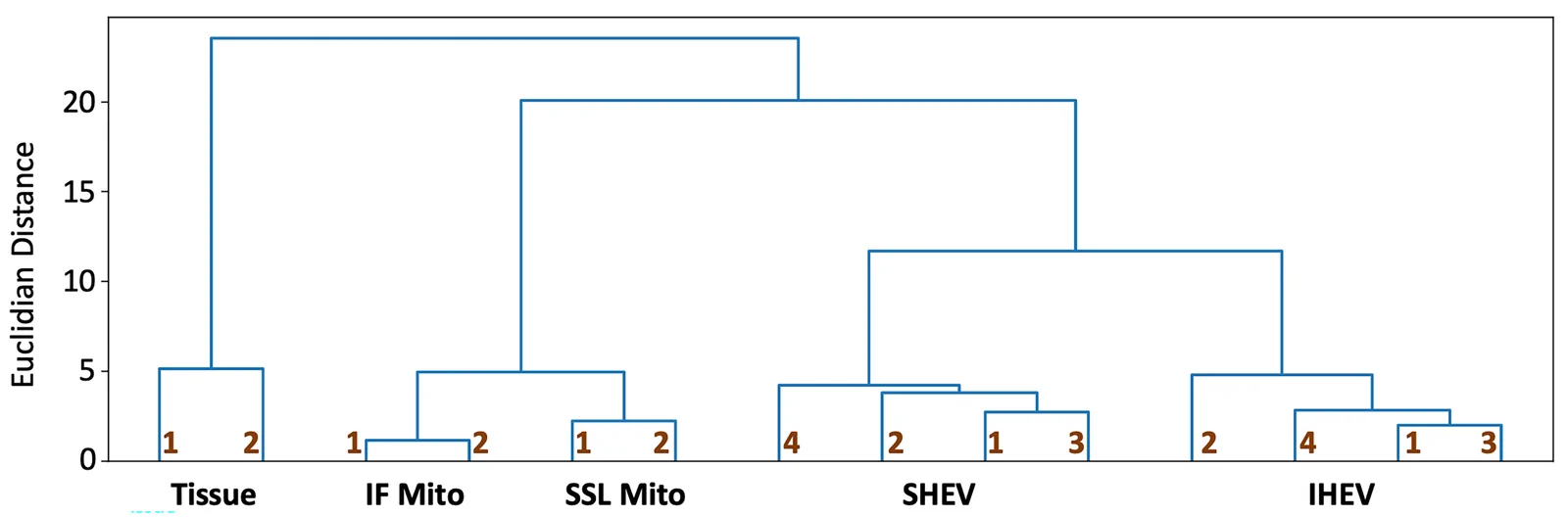
Hierarchical clustering dendrogram based on the protein content of two separate left ventricular tissue samples, IF or SSL mitochondria, or four separate SSL- or IF-associated heart EVs (SHEV, IHEV, respectively). The numbers inside the lines correspond to the patient sample numbers analyzed, as shared in Table 1. All replicates are from individual donor hearts. IF: Interfibrillar; SSL: subsarcolemmal; EV: extracellular vesicle; SHEV: subsarcolemmal heart extracellular vesicle; IHEV: interfibrillar heart extracellular vesicle.

To determine the functional profile of proteins in these vesicles, we analyzed the proteins contained within the different cellular fractions [Figure 5]. These proteins were categorized into four different functional groups plus an “other” category for proteins that did not fit into a discrete functional category. The five categories are Mitochondrial and Glycolytic (including all mitochondrial proteins); Sarcomere and Cytoskeleton; ER, Golgi, and Secretory; Vesicle Trafficking; and Other Non-Mitochondrial. The top five proteins in each category for SHEV and IHEV are listed in descending order in Table 3.

**Figure 5:**
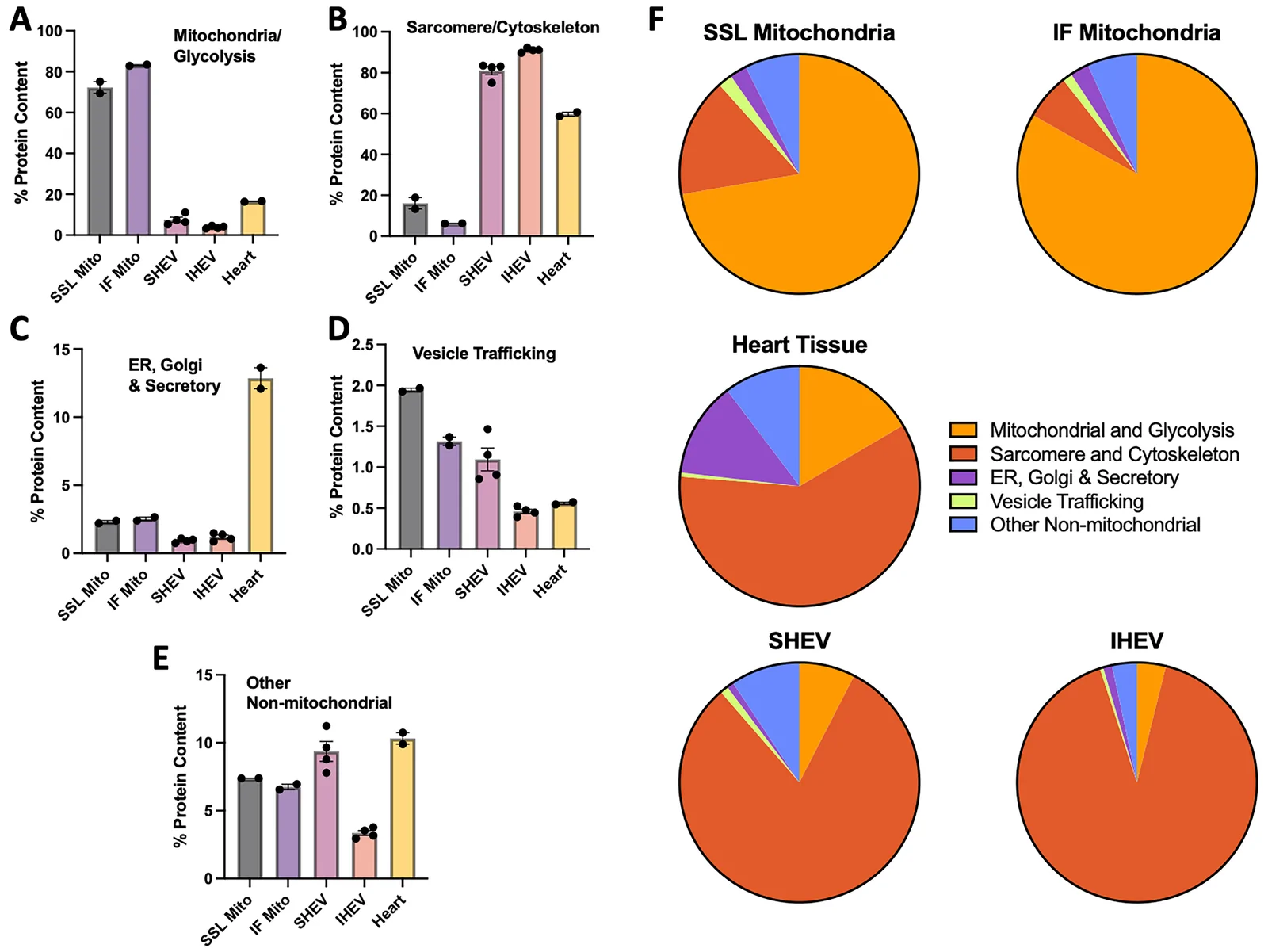
Proteins categorized by function. Percentage of detected proteins by function: (A) mitochondrial and glycolysis proteins; (B) sarcomere and cytoskeletal proteins; (C) ER, Golgi, and secretory proteins; (D) vesicular trafficking proteins; (E) other non-mitochondrial proteins; (F) fractional amounts for each protein function category by sample type, representing the average of two separate mitochondrial and tissue samples and four separate EV samples. Functional categories are shown in the legend by separate colors. *N* = 2-4 hearts. Error bars show SEM. Statistical differences in (A-E) were assessed using ordinary one-way ANOVA with multiple comparisons corrected using Tukey’s test. Complete results are provided in Supplementary Table 2. ER: Endoplasmic reticulum; EV: extracellular vesicle; SSL: subsarcolemmal; IF: interfibrillar; SHEV: subsarcolemmal heart extracellular vesicle; IHEV: interfibrillar heart extracellular vesicle; SEM: standard error of the mean; ANOVA: analysis of variance.

**Table 3:** The five most abundant proteins in EVs by functional grouping

	**SHEV**	**IHEV**
**Mitochondrial and glycolysis**	ADP/ATP translocase 1; Glyceraldehyde-3-phosphate dehydrogenase; ATP-dependent 6-phospho-fructokinase, muscle type; ATP synthase F1 subunit beta; ADP/ATP translocase 2	ADP/ATP translocase 1; Creatine kinase S-type, mitochondrial; ADP/ATP translocase 2; ADP/ATP translocase 3; Non-selective VDAC1
**Sarcomere and cytoskeleton**	Alpha-actin-2; Alpha-actin-3; Actin, alpha cardiac muscle 1; Alpha-actin-1; Beta-actin	Myosin heavy chain 7; Myosin heavy chain 6; Alpha-actin-2; Alpha-actin-3; Actin, alpha cardiac muscle 1
**ER Golgi and secretory**	Sarcalumenin; Galectin-1; Laminin subunit alpha-2; Decorin; Prolargin	Sarcalumenin; Collagen alpha-2(VI) chain; Fibrillin-1; Collagen alpha-3(VI) chain; Basement membrane-specific heparan sulfate proteoglycan core protein
**Vesicle**	Annexin A2; Clathrin heavy chain 1; EH domain-containing protein 4; Prosaposin; EH domain-containing protein 2	Annexin A2; Prosaposin; Basigin; Acid ceramidase; Prenylcysteine oxidase 1
**Other**	Alpha-crystallin B chain; Hemoglobin subunit delta; Hemoglobin subunit gamma-1; Hemoglobin subunit gamma-2; Immunoglobulin heavy constant gamma 1	Integrin alpha-9; Hemoglobin subunit delta; Endoplasmic reticulum chaperone BiP (HSP70 family protein 5); Creatine kinase B-type; VEGFR-1

EV: Extracellular vesicle; VDAC1: voltage-dependent anion-selective channel 1; HSP70: heat shock 70 kDa protein family; VEGFR-1: vascular endothelial growth factor receptor 1.

### Phospholipid profile

Phospholipids, especially CL, are essential for proper mitochondrial function. Furthermore, since CL, unique among phospholipids, is found only in mitochondria, its presence serves as a marker of mitochondrial-derived membranes^[17]^. Therefore, we determined the profile of 8 different phospholipids, including CL, from 2 of the hearts for which we had EV, mitochondria, and whole homogenate samples. The absolute amounts of phospholipids are shown in Figure 6, and relative amounts are expressed as a percentage of the total phospholipid content in Figure 7. IHEVs had a much greater phospholipid content (expressed per mg of protein) than SHEVs, often higher even than mitochondrial samples [Figure 6A-I]. The relative amounts of different phospholipids in the two vesicle types were similar, with a higher percentage of SM than in mitochondria or tissue and higher PA and PG than in tissue. SHEVs had a higher percentage of PE than SSL mitochondria and a lower percentage of CL [Figure 7]. CL molecular species are defined as having a unique number of carbons and double bonds on their four fatty acyl side chains. CL molecular species are often altered in response to stress and disease, but it is unknown whether EVs would be enriched for certain CL species. Figure 8 shows the percent distribution of the nine most abundant CL species in mitochondria, EVs, and tissue. Most species were not significantly different between EVs and mitochondria or tissue, except for two higher-molecular-weight species [Figure 8G and H]. These data indicate that there is a conversion between the CL species having side chains (carbons: double bonds) of 76:12 to 74:9, resulting in a loss of 2 carbons and 3 double bonds on CL.

**Figure 6:**
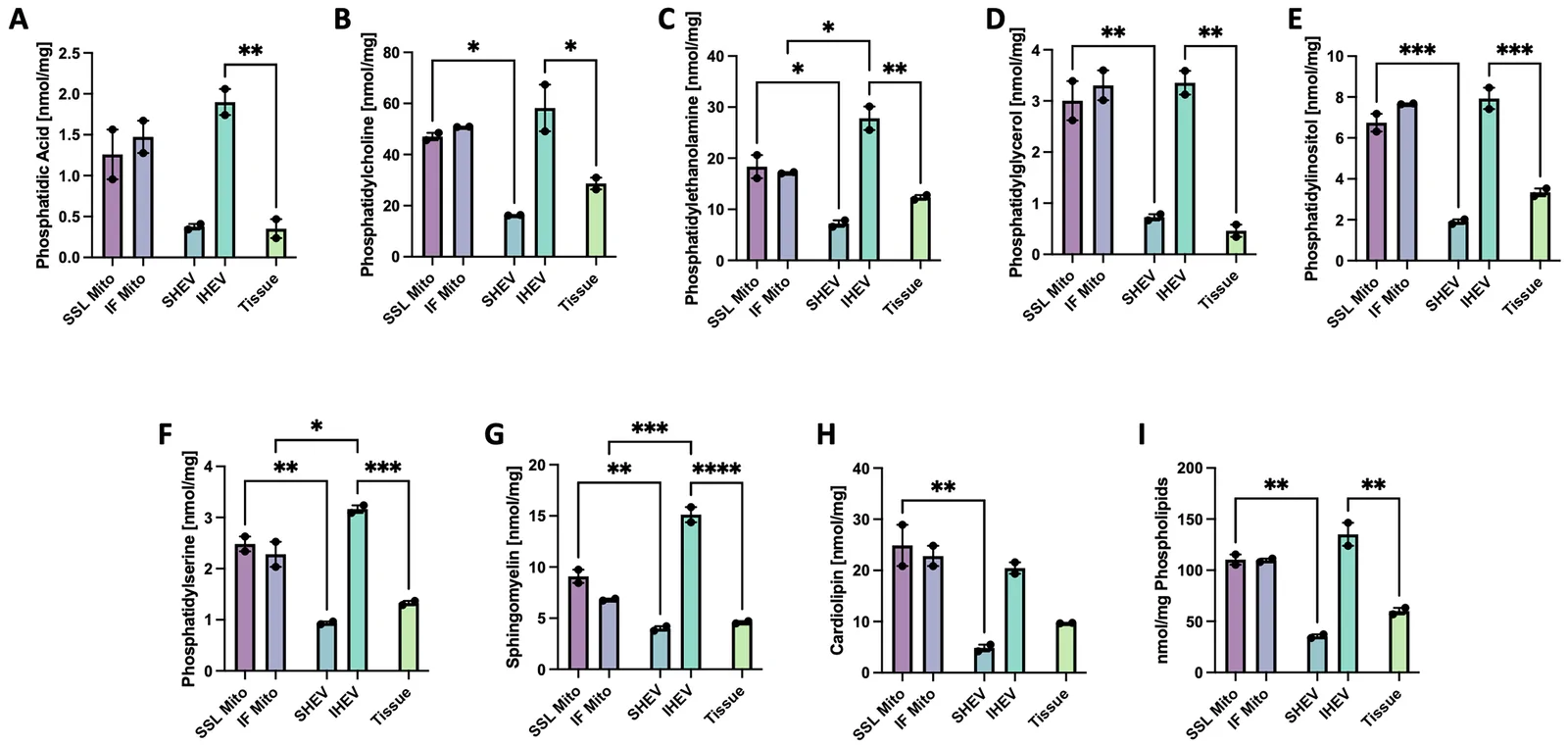
Absolute amounts in nmol/mg protein of eight phospholipid classes and their sum in SSL or IF mitochondria, SSL- or IF-associated heart EVs (SHEV and IHEV, respectively), or left ventricular tissue. Graphs show the sum of detected species for (A) PA, (B) PC, (C) PE, (D) PG, (E) PI, (F) PS, (G) SM, (H) CL, and (I) sum of all phospholipid species. ^*^P < 0.05, ^**^P < 0.01, ^***^P < 0.001, ^****^P < 0.0001; *N* = 2 donor hearts. Because of the limited biological sample size (*N* = 2), these data are presented as exploratory/descriptive findings. Individual donor values are shown. Error bars show SEM. Analysis was performed using ordinary one-way ANOVA with multiple comparisons corrected using Tukey’s test. Comparisons in this dataset were SSL Mito *vs.* SHEV, IF Mito *vs.* IHEV, SHEV *vs.* Tissue, and IHEV *vs.* Tissue. SSL: Subsarcolemmal; IF: interfibrillar; EV: extracellular vesicle; SHEV: subsarcolemmal heart extracellular vesicle; IHEV: interfibrillar heart extracellular vesicle; PA: phosphatidic acid; PC: phosphatidylcholine; PE: phosphatidylethanolamine; PG: phosphatidylglycerol; PI: phosphatidylinositol; PS: phosphatidylserine; SM: sphingomyelin; CL: cardiolipin; SEM: standard error of the mean; ANOVA: analysis of variance.

**Figure 7:**
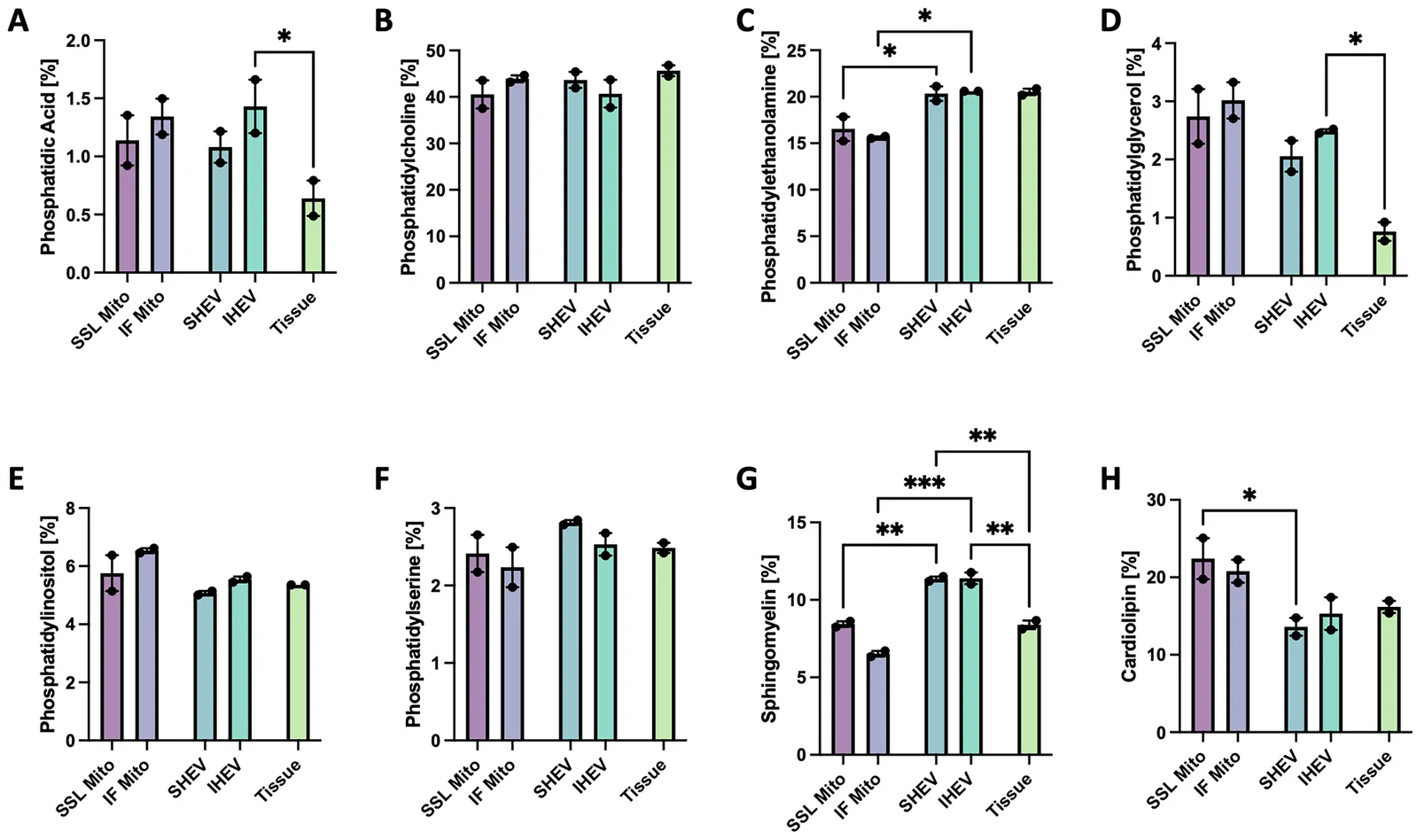
Percentage of eight phospholipid classes in SSL or IF mitochondria, SSL- or IF-associated heart EVs (SHEV, IHEV, respectively), or left ventricular tissue. Graphs show the percentage of the sum of detected species for (A) PA, (B) PC, (C) PE, (D) PG, (E) PI, (F) PS, (G) SM, and (H) CL. ^*^P < 0.05, ^**^P < 0.01, ^***^P < 0.001; *N* = 2 donor hearts. Because of the limited biological sample size (*N* = 2), these data are presented as exploratory/descriptive findings. Individual donor values are shown. Error bars show SEM. Analysis was performed using ordinary one-way ANOVA with multiple comparisons corrected using Tukey’s test. Comparisons in this dataset were between SSL Mito and SHEV, IF Mito and IHEV, SHEV and Tissue, and IHEV and Tissue. SSL: Subsarcolemmal; IF: interfibrillar; EV: extracellular vesicle; SHEV: subsarcolemmal heart extracellular vesicle; IHEV: interfibrillar heart extracellular vesicle; PA: phosphatidic acid; PC: phosphatidylcholine; PE: phosphatidylethanolamine; PG: phosphatidylglycerol; PI: phosphatidylinositol; PS: phosphatidylserine; SM: sphingomyelin; CL: cardiolipin; SEM: standard error of the mean; ANOVA: analysis of variance.

**Figure 8:**
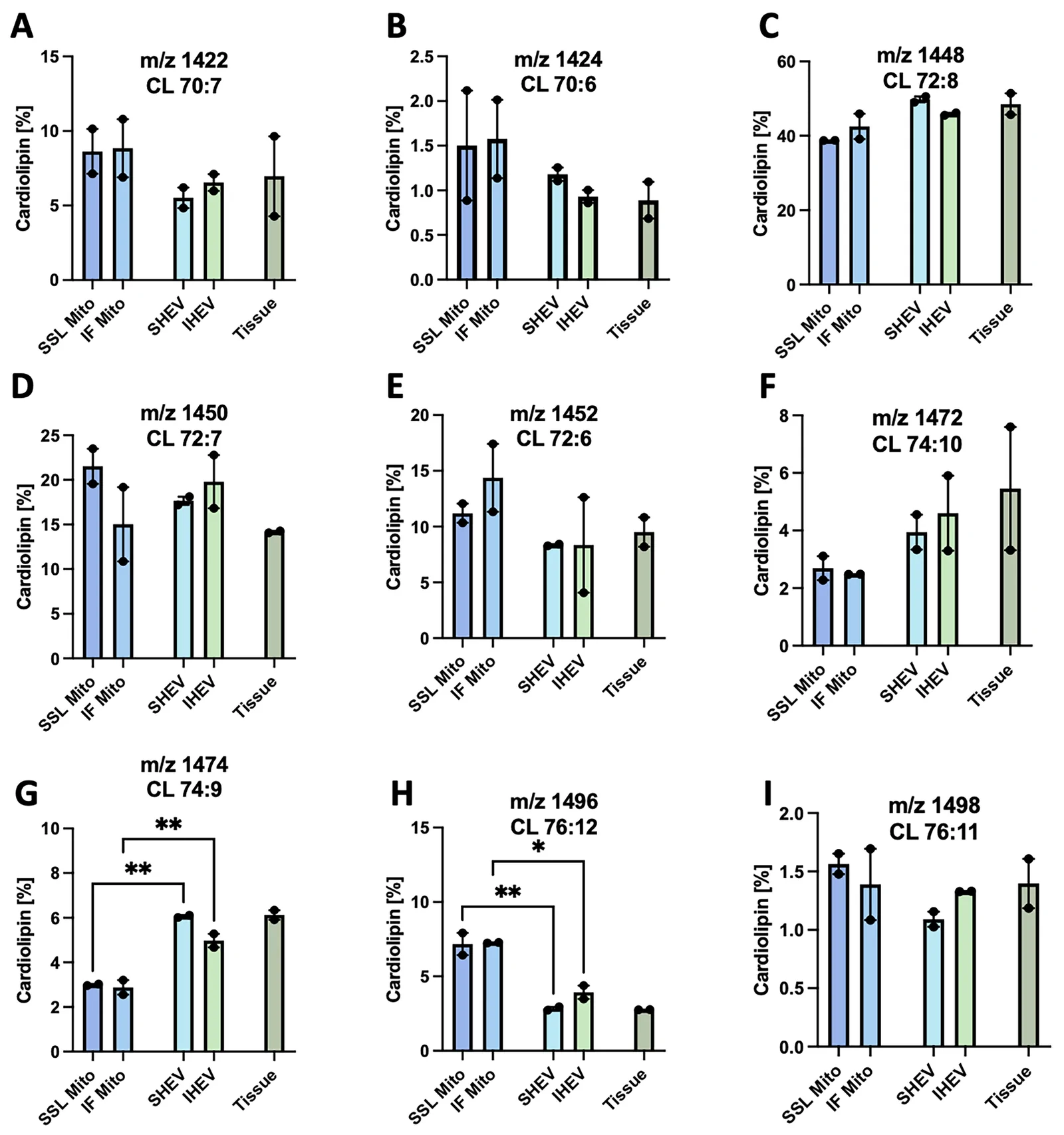
Percentage of individual CL species in SSL or IF mitochondria, SSL- or IF-associated heart EVs (SHEV, IHEV, respectively), or left ventricular tissue. Graphs show the percentage of the sum of the nine most prevalent CL species by mass/charge ratio (m/z) and number of side-chain carbons:double bonds for (A) 1422, 70:7; (B) 1424, 70:6; (C) 1448, 72:8; (D) 1450, 72:7; (E) 1452, 72:6; (F) 1472, 74:10; (G) 1474, 74:9; (H) 1496, 76:12; and (I) 1498, 76:11. ^*^P < 0.05, ^**^P < 0.01; *N* = 2 donor hearts. Because of the limited biological sample size (*N* = 2), these data are presented as exploratory/descriptive findings. Individual donor values are shown. Error bars show SEM. Analysis was performed using ordinary one-way ANOVA with multiple comparisons corrected using Tukey’s test. Comparisons in this dataset were SSL Mito vs SHEV, IF Mito vs IHEV, SHEV vs Tissue, and IHEV vs Tissue. CL: Cardiolipin; m/z: mass-to-charge; SSL: subsarcolemmal; IF: interfibrillar; EV: extracellular vesicle; SHEV: subsarcolemmal heart extracellular vesicle; IHEV: interfibrillar heart extracellular vesicle; SEM: standard error of the mean; ANOVA: analysis of variance.

### Functional assessment of mitochondrial activity

To determine whether SHEVs or IHEVs affect the function of cardiomyocyte mitochondria or have an effect on their own, a standardized dose of 5 × 10^8^ EV particles was added to primary NRVMs (1 × 10^4^ particles per plated cell) for 48 h, a standard length of time in our laboratory to produce an effect on mitochondria, and the mitochondrial function of NRVMs was assessed using the Seahorse Bioanalyzer. SHEV treatment increased ATP production and basal respiration relative to untreated NRVMs, whereas IHEV treatment decreased maximal respiration [Figure 9]. SHEV- and IHEV-associated responses also differed significantly in ATP production, basal respiration, and maximal respiration. Although maximal respiration tended to increase following SHEV treatment, this change did not reach statistical significance. Importantly, SHEV and IHEV preparations added to Seahorse wells in the absence of NRVMs exhibited no detectable oxygen consumption, indicating that the EV preparations did not contribute measurable intrinsic respiratory activity under these assay conditions [Supplementary Figure 6]. The principal bioenergetic responses were reproduced in a second independent NRVM isolation using the same donor-derived EV preparations [Supplementary Figure 7]. CyQUANT measurements obtained following 48 h EV treatment showed no significant reduction in viable-cell abundance in either SHEV- or IHEV-treated NRVMs compared with untreated controls [Supplementary Figure 8].

**Figure 9:**
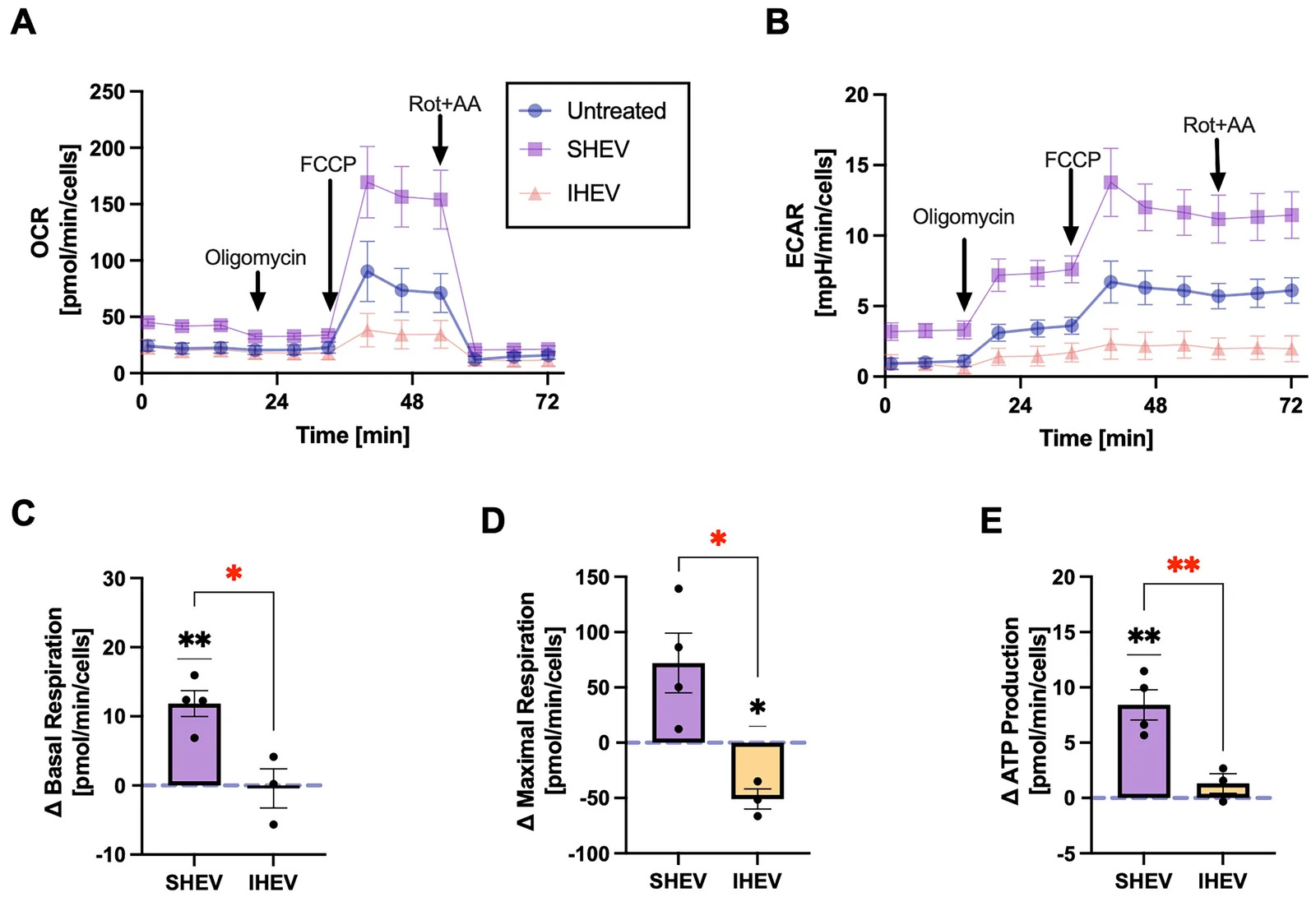
Effects of particle-normalized SHEV and IHEV treatment on cardiomyocyte mitochondrial bioenergetics. (A) OCR and (B) ECAR in untreated cardiomyocytes and cardiomyocytes treated with SHEVs or IHEVs. Arrows indicate substrate or inhibitor additions; (C) change in basal respiration; (D) maximal respiration; and (E) ATP production relative to the mean untreated response from the same NRVM preparation (Δ = EV-treated donor mean - untreated mean). SHEV and IHEV preparations were administered at 5 × 10^8^ particles/well, corresponding to approximately 1 × 10^4^ particles per plated cell. Each point in (C-E) represents one independently derived human EV donor preparation and is the mean of approximately five technical Seahorse wells (SHEV, *n* = 4 donor hearts; IHEV, *n* = 3 donor hearts). The dashed horizontal line at Δ = 0 represents the untreated NRVM reference. Donor-level Δ values were compared with zero using two-sided one-sample *t*-tests; black significance symbols indicate comparisons with untreated. SHEV *vs*. IHEV comparisons were performed using Welch’s unpaired *t*-test and are indicated by red significance symbols. ^*^P < 0.05; ^**^P < 0.01. Error bars indicate SEM. SHEV: Subsarcolemmal heart extracellular vesicle; IHEV: interfibrillar heart extracellular vesicle; OCR: oxygen consumption rate; ECAR: extracellular acidification rate; ATP: adenosine triphosphate; NRVM: neonatal rat ventricular myocyte; EV: extracellular vesicle; SEM: standard error of the mean.

## DISCUSSION

Although cardiac EVs have been documented, to our knowledge, this is the first study to isolate and characterize human cardiac EVs associated with specific mitochondrial subtypes. Our data support regionally distinct EV populations with reproducible molecular and functional differences. In addition, because fresh, nonfailing human hearts are unavailable, most reports in the literature use rodents rather than human heart tissue. Since mitochondria ideally need to be isolated from fresh tissue for the highest possible yield and purity, studies of mitochondria isolated from human hearts are virtually nonexistent outside of a few laboratories^[18]^. While the presence of disease has been reported to change the secretion/release and the cargo of cardiac EVs^[19]^, our current study used nonfailing donor hearts to establish a baseline for further studies, as tissue becomes available, that will include diseased hearts. The approach in this study was to isolate both cardiac surface vesicles, SHEVs, and vesicles originating from the interior of the cardiomyocyte, IHEVs, assess their protein and phospholipid content, including the identities and functions of the proteins and phospholipids associated with mitochondria, and determine their direct effect on mitochondrial function in primary cardiomyocytes. We found that the SHEVs and IHEVs contained different amounts of cargo, with metabolic (mitochondrial and glycolytic) proteins making up 7.6% of protein content in SHEVs and 3.9% in IHEVs. In contrast, the IHEVs contained a higher concentration of contractile and structural proteins, which makes sense given they are derived from the region of the heart that contains the contractile machinery. These functional categorizations of proteins were performed in EVs from four separate hearts, and the percentages are shown in Figure 5. Despite the variability typically introduced by differences among individual donors, the EVs isolated from all hearts exhibited a remarkably consistent profile. In addition, the absolute levels of several phospholipids were much higher relative to protein in the IHEVs (PE, SM, PS), with all phospholipids being lower in the SHEVs compared to isolated mitochondria or heart tissue. The percentage of each phospholipid in the EVs was close to that found in mitochondria, except for PE and SM, which were both higher in the vesicles. Interestingly, the mitochondrial phospholipid CL was the only phospholipid identified as lower in EVs than in mitochondria, consistent with CL being expressed specifically in mitochondria. This may also point to a lack of secretion in healthy, non-failing hearts, raising the question of whether it is present in EVs from failing hearts. We will investigate this further as tissue becomes available. In addition, the percentage of all eight phospholipids was not significantly different between SHEVs and IHEVs, indicating that these vesicles had similar, but unique, phospholipid profiles that are also distinct for either mitochondria or tissue. In vesicles, a given phospholipid molecule can constitute either cargo or membrane, with previous studies showing enrichment of phospholipids that were dependent on EV origin and type, with SM and PE tending to be higher in most vesicle types^[20,21]^. Given the bulk lipid isolation methods used for phospholipid analysis in our current study, and the limited sample availability, we did not distinguish where these phospholipids reside (i.e., surface *vs*. inner cargo of the EVs). Since the location of these phospholipids can affect their function, this is planned for future studies comparing diseased *vs*. nonfailing heart tissue. Within the CL profile, we detect a conversion in both EV types between the CL species with 76 carbons and 12 double bonds (m/z 1496) and 74 carbons and 9 double bonds (m/z 1474), resulting in a loss of 2 carbons and 3 double bonds in the CL profile. This could occur if arachidonic acid (AA; 20:4n6) is substituted for docosahexaenoic acid (DHA; 22:6n3), resulting in a loss of 2 carbons and 2 fatty acids, plus another substitution of linoleic acid (LA; 18:2n6) with oleic acid (OA; 18:1n9) for the loss of one double bond. The four acyl side chains of m/z 1496 CL (DHA_1_LA_3_) and m/z 1474 (AA_1_OA_1_LA_2_) CL molecules make this a likely scenario. The question is then why the substitution occurs. A recent study that looked at changes between EV fatty acids and the cells they were derived from did see an increase in oleic acid and arachidonic acid, a general desaturation of fatty acids, and a decrease in the n3/n6 fatty acid levels, all of which fit with the shifts we see in the CL profile^[21]^.

In this study, a very low amount of trypsin (1 mg trypsin/g tissue) was used to dislodge the IF mitochondria from the myofibrils. In contrast, over 1,000 times more trypsin per mg of EV tissue was used to study EV proteomics in a recent publication^[22]^. The trypsin levels used in our current study should therefore not affect the proteomic profiles obtained and likely do not affect vesicle functionality, especially since IF mitochondria are routinely isolated this way and then used functionally for a wide range of studies.

Because primary human heart cells are not routinely cultured and human pluripotent stem cell-derived cardiomyocytes often lose their mitochondrial substrate preference in culture, rat cardiomyocytes (NRVMs) are commonly used as a substitute when cardiac acceptor cells are required. Our laboratory has shown that NRVMs treated with serum from human patients with heart failure serve as a valuable model to study mechanisms of heart failure^[23]^. This is interesting since serum contains EVs.

SHEVs and IHEVs have opposite effects on acceptor cells: SHEVs increase ATP production, basal respiration, and maximal respiration, whereas IHEVs reduce maximal respiration without altering ATP production or basal respiration and, importantly, without affecting recipient-cell viability. These divergent responses demonstrate that the two EV-enriched populations differentially influence cardiomyocyte bioenergetics. The molecular mechanisms responsible for these effects remain unknown and cannot be determined from the present experiments. Future studies examining EV uptake, cargo-dependent signaling, and responses under disease-relevant stress conditions are needed to define their mechanisms of action.

To address whether these vesicles are purely mitochondrially derived (MDVs) is not straightforward, given that not every protein made in a cell or in a mitochondrion will be placed in or on a vesicle. Our findings indicate that less than 10% of the protein in or on SHEVs and IHEVs is of mitochondrial origin, and SHEVs can increase mitochondrial basal respiration and ATP production in rat heart cells. This could point to more direct transfer of mitochondrial cargo from SHEVs to cells, or it could result from other cargo, not assessed in this work, modifying these activities. This finding is of great interest as it points to the possibility that in the heart during stress, disease, or response to treatments such as doxorubicin, the cargo in the SHEVs may be altered to become more mitochondrial and/or contain specific proteins like the ATPase cargo found in yeast that can in turn maintain basal respiration and ATP production in a failing heart^[4,24]^. Ongoing work will determine how these observations change in the human heart under disease conditions.

### Limitations of this study

The primary limitation of this study is the limited availability of fresh nonfailing human donor hearts, which restricted sample size for several analyses, particularly the proteomic and lipidomic comparisons. Accordingly, these findings should be interpreted as descriptive, establishing regionally distinct EV populations in the human heart and their associated molecular and functional differences. Larger studies will be required to confirm these observations across a broader donor population. In addition, tissue samples were obtained from the free wall of the left ventricle after removal of epicardial fat. Because intact ventricular tissue was processed rather than anatomically separated myocardial layers or purified cell populations, we cannot determine the relative contributions of epicardial *vs*. endocardial tissue or definitively assign the cellular origin of the EVs. Finally, EVs were enriched using a precipitation-based separation method to maximize recovery from the limited amount of available human tissue. While this approach enabled comprehensive downstream molecular and functional analyses, it may also enrich non-EV particles or other extracellular components. Future studies using orthogonal isolation methods, together with additional characterization and diseased tissue, will further refine these observations. Despite these limitations, the molecular profiles and functional responses were highly consistent across independently derived donor samples, supporting the reproducibility of the observed regional differences.

### Conclusion

This study identifies regionally distinct EV populations in the nonfailing human heart that differ in protein and phospholipid composition and in their effects on cardiomyocyte mitochondrial bioenergetics. SHEVs enhanced cardiomyocyte bioenergetic function, whereas IHEVs reduced maximal respiratory capacity, even though neither EV population showed intrinsic respiratory activity in the absence of cardiomyocytes. Together, these findings support a previously unrecognized role for region-specific EV-mediated communication in the human heart and provide a foundation for determining how these EV populations and their functions are altered in cardiac disease.

## DECLARATIONS

### Availability of data and materials

The raw data supporting the findings of this study are available within this Article and its Supplementary Materials (Proteomics Eight EVs.xlsx; Proteomics whole tissue.xlsx; Proteomics mitos.xlsx; Cardiolipin Data.xlsx; Phospholipid Data.xlsx; Seahorse Data July 2026.xlsm). The corresponding authors can provide additional data upon request.

### AI and AI-assisted tools statement

During the preparation of this manuscript, the AI tool Claude Science (Claude Science Beta Version 0.1.24, 2026-06-30) was used to classify proteins in the proteomics dataset, to generate the hierarchical clustering dendrogram in Figure 4, and to perform the analyses presented in Supplementary Figure 4. The tool did not influence the study design, data collection, analysis, interpretation, or the scientific content of the work. All authors take full responsibility for the accuracy, integrity, and final content of the manuscript.

### Copyright

© The Author(s) 2026.

## References

[1] Bleck CKE, Kim Y, Willingham TB, Glancy B (2018). Subcellular connectomic analyses of energy networks in striated muscle. Nat Commun.

[2] Palmer J, Tandler B, Hoppel C (1977). Biochemical properties of subsarcolemmal and interfibrillar mitochondria isolated from rat cardiac muscle. J Biol Chem.

[3] Riva A, Tandler B, Loffredo F, Vazquez E, Hoppel C (2005). Structural differences in two biochemically defined populations of cardiac mitochondria. Am J Physiol Heart Circ Physiol.

[4] Cadete VJ, Deschênes S, Cuillerier A (2016). Formation of mitochondrial-derived vesicles is an active and physiologically relevant mitochondrial quality control process in the cardiac system. J Physiol.

[5] Picca A, Guerra F, Calvani R (2023). Mitochondrial-derived vesicles: the good, the bad, and the ugly. Int J Mol Sci.

[6] Heyn J, Heuschkel MA, Goettsch C (2023). Mitochondrial-derived vesicles-link to extracellular vesicles and implications in cardiovascular disease. Int J Mol Sci.

[7] Mishra S, Deep G (2024). Mitochondria-derived vesicles: potential nano-batteries to recharge the cellular powerhouse. Extracell Vesicles Circ Nucl Acids.

[8] Veksler VI, Kuznetsov AV, Sharov VG, Kapelko VI, Saks VA (1987). Mitochondrial respiratory parameters in cardiac tissue: a novel method of assessment by using saponin-skinned fibers. Biochim Biophys Acta.

[9] McCabe MC, Schmitt LR, Hill RC (2021). Evaluation and refinement of sample preparation methods for extracellular matrix proteome coverage. Mol Cell Proteomics.

[10] Kong AT, Leprevost FV, Avtonomov DM, Mellacheruvu D, Nesvizhskii AI (2017). MSFragger: ultrafast and comprehensive peptide identification in mass spectrometry-based proteomics. Nat Methods.

[11] Chitti SV, Gummadi S, Kang T (2024). Vesiclepedia 2024: an extracellular vesicles and extracellular particles repository. Nucleic Acids Res.

[12] Sucharov CC, Mariner PD, Nunley KR, Long C, Leinwand L, Bristow MR (2006). A beta1-adrenergic receptor CaM kinase II-dependent pathway mediates cardiac myocyte fetal gene induction. Am J Physiol Heart Circ Physiol.

[13] Li H, Liu Y, Lin Y (2025). Cardiac repair using regenerating neonatal heart tissue-derived extracellular vesicles. Nat Commun.

[14] Vandergriff A, Huang K, Shen D (2018). Targeting regenerative exosomes to myocardial infarction using cardiac homing peptide. Theranostics.

[15] Yang J, Yun X, Zheng W (2025). Nanoscale engineered exosomes for dual delivery of Sirtuin3 and insulin to ignite mitochondrial recovery in myocardial ischemia-reperfusion. J Nanobiotechnology.

[16] Rath S, Sharma R, Gupta R (2021). MitoCarta3.0: an updated mitochondrial proteome now with sub-organelle localization and pathway annotations. Nucleic Acids Res.

[17] Ren M, Phoon CK, Schlame M (2014). Metabolism and function of mitochondrial cardiolipin. Prog Lipid Res.

[18] Cordero-Reyes AM, Gupte AA, Youker KA (2014). Freshly isolated mitochondria from failing human hearts exhibit preserved respiratory function. J Mol Cell Cardiol.

[19] Fu S, Zhang Y, Li Y, Luo L, Zhao Y, Yao Y (2020). Extracellular vesicles in cardiovascular diseases. Cell Death Discov.

[20] Haraszti RA, Didiot MC, Sapp E (2016). High-resolution proteomic and lipidomic analysis of exosomes and microvesicles from different cell sources. J Extracell Vesicles.

[21] Mustonen AM, Paakkonen T, Matilainen J (2022). Fatty acid fingerprints and hyaluronic acid in extracellular vesicles from proliferating human fibroblast-like synoviocytes. Int J Mol Sci.

[22] Choi D, Go G, Kim DK (2020). Quantitative proteomic analysis of trypsin-treated extracellular vesicles to identify the real-vesicular proteins. J Extracell Vesicles.

[23] Jeffrey DA, Pires Da Silva J, Garcia AM (2021). Serum circulating proteins from pediatric patients with dilated cardiomyopathy cause pathologic remodeling and cardiomyocyte stiffness. JCI Insight.

[24] Hazan Ben-Menachem R, Lintzer D, Ziv T (2023). Mitochondrial-derived vesicles retain membrane potential and contain a functional ATP synthase. EMBO Rep.

