# Supplementary material for: Region-specific human cardiac extracellular vesicles differentially regulate mitochondrial function
Source: Extracell Vesicles Circ Nucl Acids. 2026 Sep 28;7(3):1582–99. doi: 10.20517/evcna.2026.58 (PMC13673452; doi:10.20517/evcna.2026.58)
Supplement: Supplementary file 1 [file evcna-7-3-1582-SupplementaryMaterials.pdf]

Supplementary Materials

Region-specific human cardiac extracellular vesicles differentially regulate mitochondrial function

Genevieve C. Sparagna[1,#], Christina M. Coughlan[2,#], Hailey L. Chapman[3], Sydney R. Shuff[1], Ashley E. Pietra[4], Benjamin J. Kopecky[1], Alicia J. Chapman[1], Eva M. Medina[5], Anthony J. Saviola[5], Kathryn C. Chatfield[4], Shelley D. Miyamoto[4,†], Anastacia M. Garcia[4,†]

[1]Department of Medicine, Division of Cardiology, University of Colorado Anschutz Medical Campus, Aurora, CO 80045, USA.
[2]Department of Neurology, University of Colorado Anschutz, Aurora, CO 80045, USA.
[3]Department of Life Sciences, Division of Systems and Synthetic Biology, Chalmers University of Technology, Gothenburg 41296, Sweden.
[4]Department of Pediatrics, Division of Cardiology, University of Colorado Anschutz Medical Campus and Children's Hospital Colorado, Aurora, CO 80045, USA.
[5]Department of Biochemistry and Molecular Genetics, University of Colorado Anschutz Medical Campus, Aurora, CO 80045, USA.
[#]These authors are joint first authors.
[†]These authors are joint senior authors.

Correspondence to: Dr. Christina M. Coughlan, Department of Neurology, University of Colorado Anschutz, Aurora, CO 80045, USA. E-mail: christina.coughlan@cuanschutz.edu

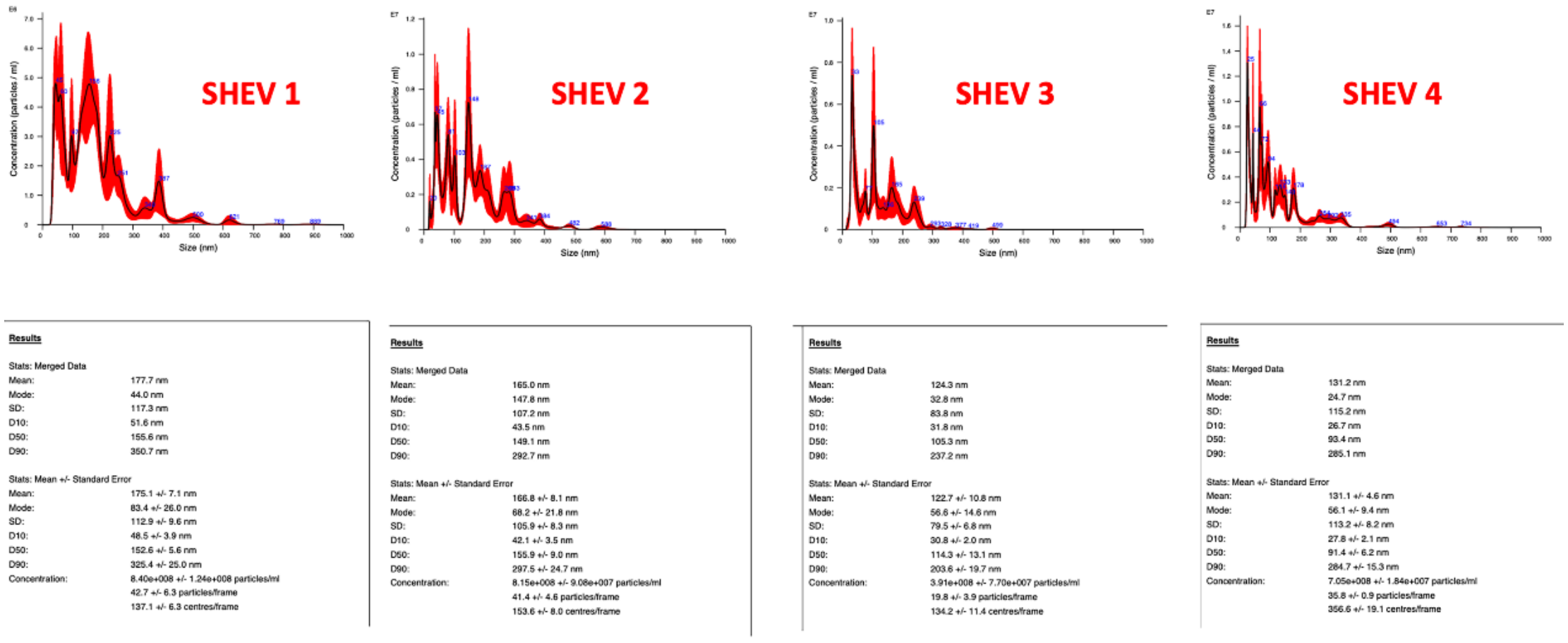

Supplementary Figure 1. Nanosight average curves from SHEV preparations from four donors. Numbers correspond to the donors in Tables 1 and 2. SHEV: Subsarcolemmal heart extracellular vesicle.

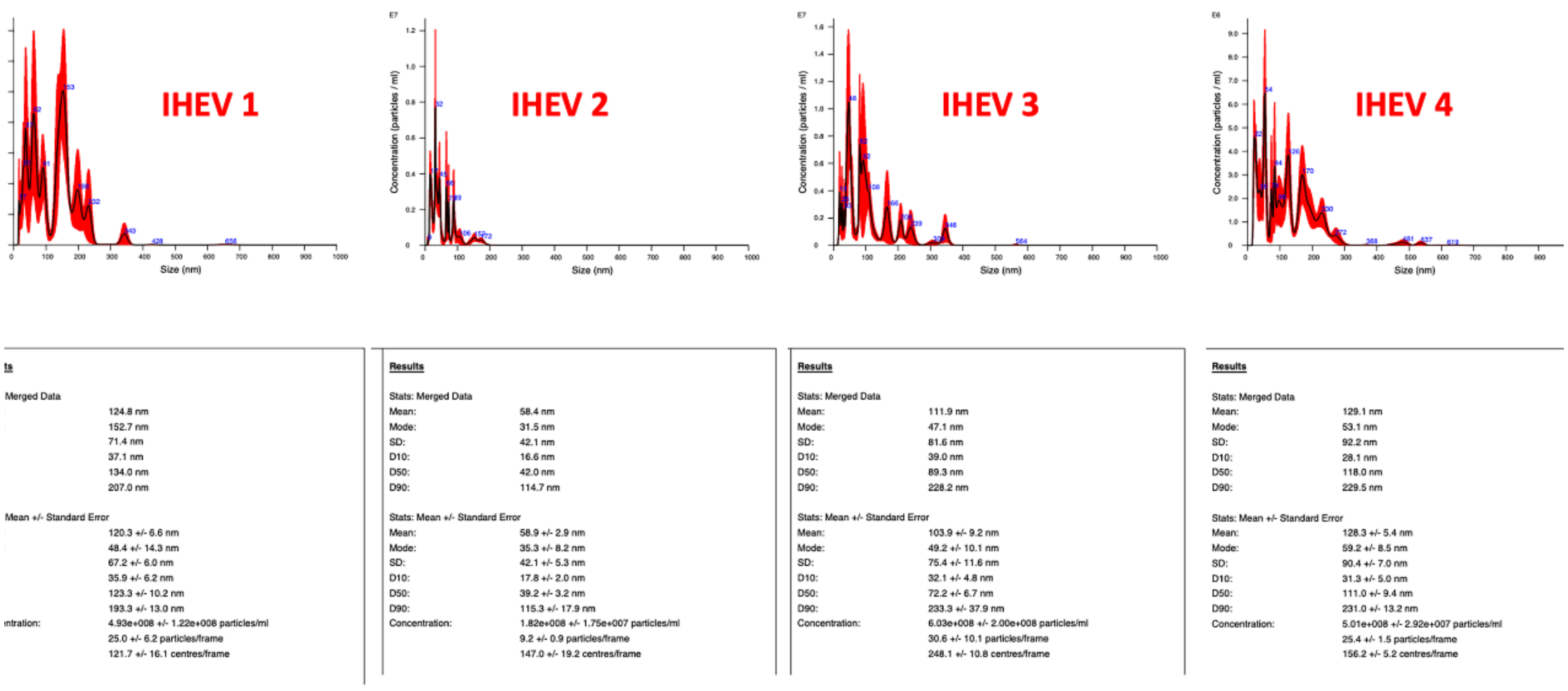

Supplementary Figure 2. Nanosight average curves from IHEV preparations from four donors. Numbers correspond to the donors in Tables 1 and 2. IHEV: Interfibrillar heart extracellular vesicle.

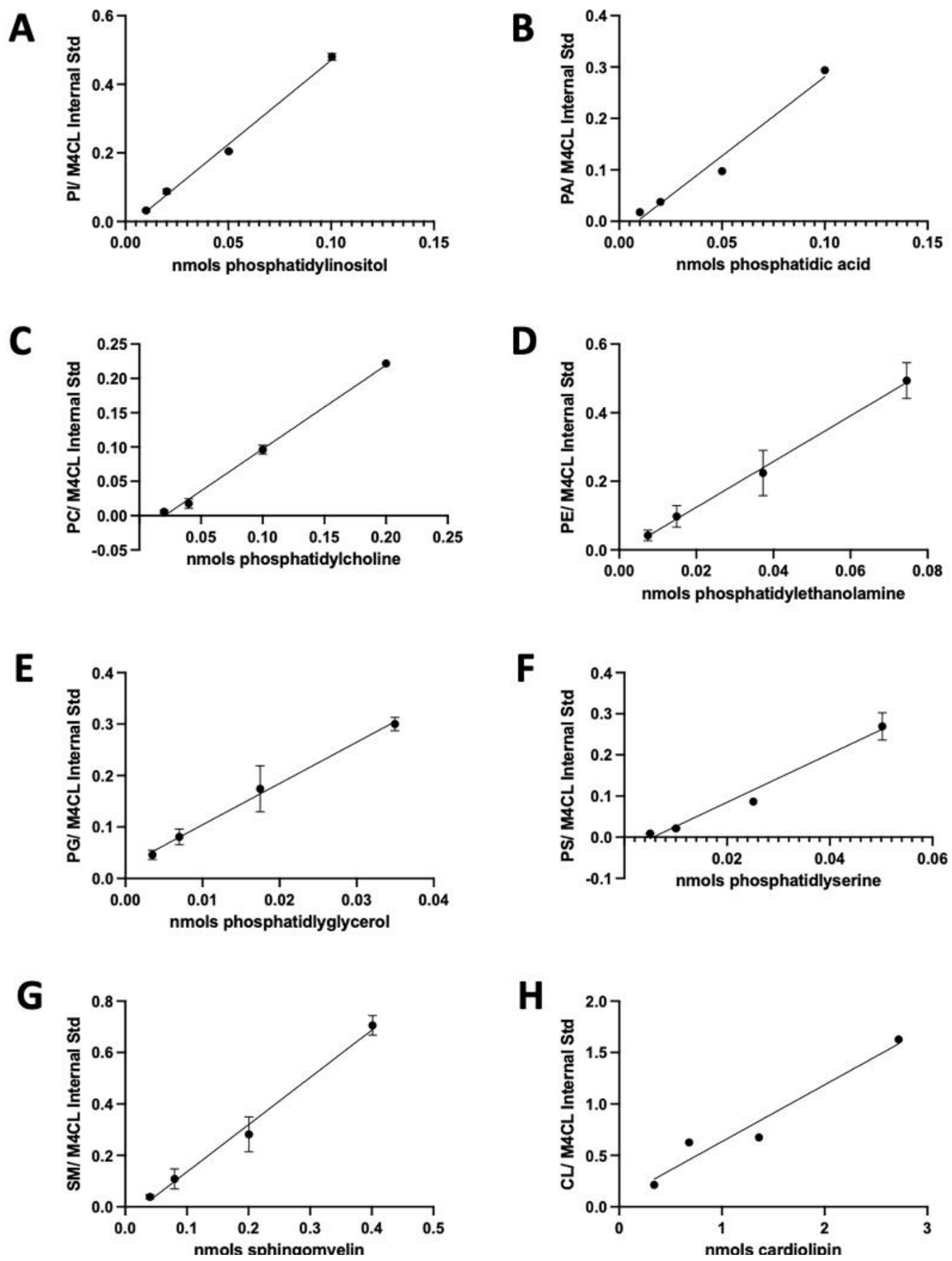

I

	slope	intercept
PG	4.22	0.28
PE	6.67	-0.01
PI	4.90	-0.02
PS	5.87	-0.03
PA	2.89	0.00
PC	1.23	-0.03
SM	1.84	-0.05
CL	0.55	0.08

Supplementary Figure 3. Standard curves, slopes, and intercepts for each of the eight phospholipids against tetramyristoylcardiolipin. (A) PI; (B) PA; (C) PC; (D) PE; (E) PG; (F) PS; (G) SM; (H) CL; (I) slopes and intercepts of the curves in (A–H). PI: Phosphatidylinositol; PA: phosphatidic acid; PC: phosphatidylcholine; PE: phosphatidylethanolamine; PG: phosphatidylglycerol; PS: phosphatidylserine; SM: sphingomyelin; CL: cardiolipin.

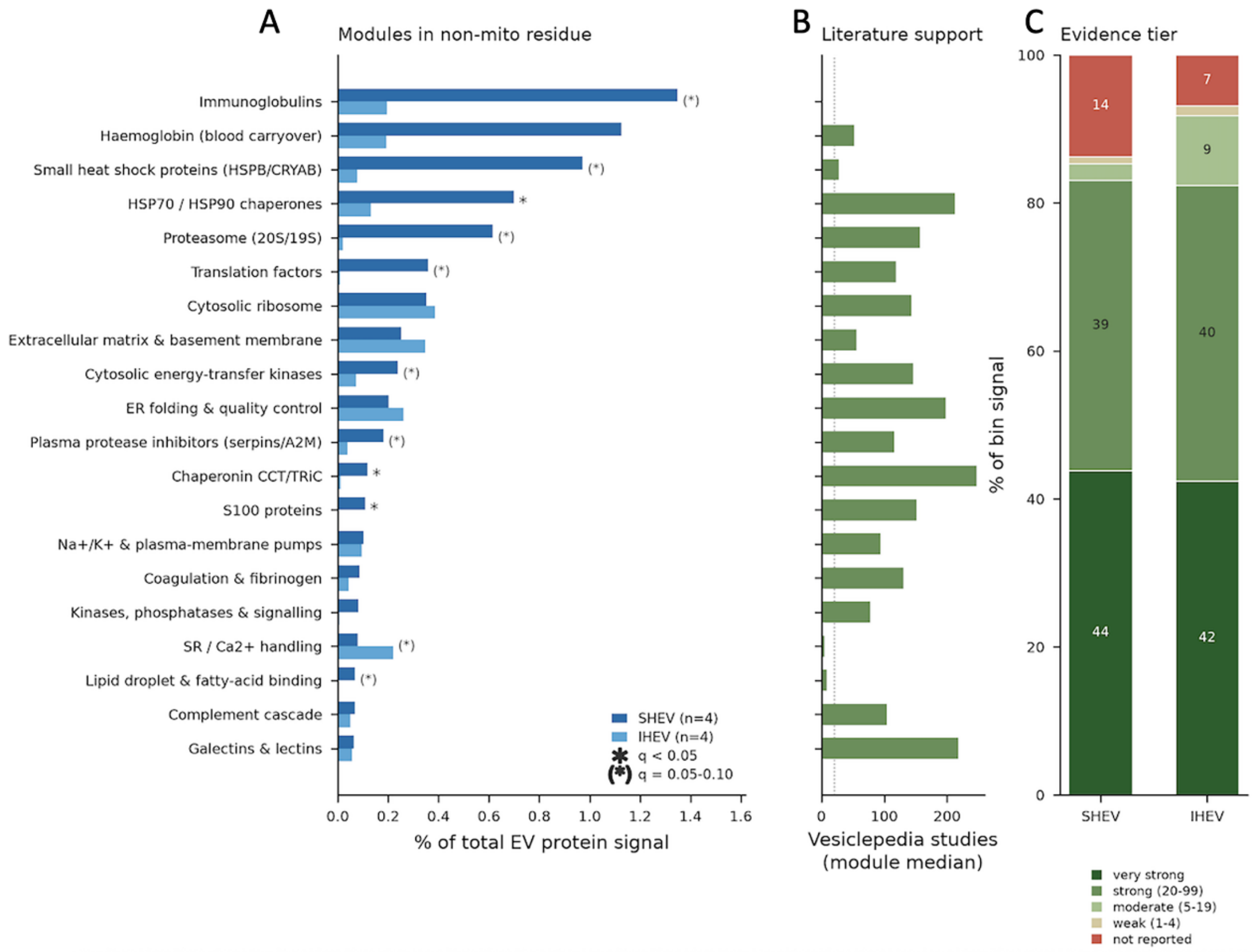

Supplementary Figure 4. Protein modules in the "Other Non-mitochondrial" fraction of cardiac EV proteomes, with EV-literature support as analyzed by Claude Science. (A) Summed abundance of each functional module in the "Other Non-Mitochondrial" category as a percentage of total EV protein signal, mean of n=4 SHEV and n=4 IHEV preparations. Significance is a paired t-test of module sums across the four donor-matched SHEV/IHEV pairs, Benjamini-Hochberg corrected across all 35 modules tested: $*q < 0.05$; (*) $q = 0.05–0.10$; (B) Module median number of distinct published EV experiments reporting each member protein (Vesiclepedia 5.1, human protein records, joined on Entrez Gene ID); dotted line, 20 studies; (C) Composition of each EV type's fraction signal by per-protein evidence tier, as % of that EV type's bin total; tiers are cut on Vesiclepedia study count (very strong ⩾100; strong 20–99; moderate 5–19; weak 1–4; not reported). EV: Extracellular vesicle; SHEV: subsarcolemmal heart extracellular vesicle; IHEV: interfibrillar heart extracellular vesicle.

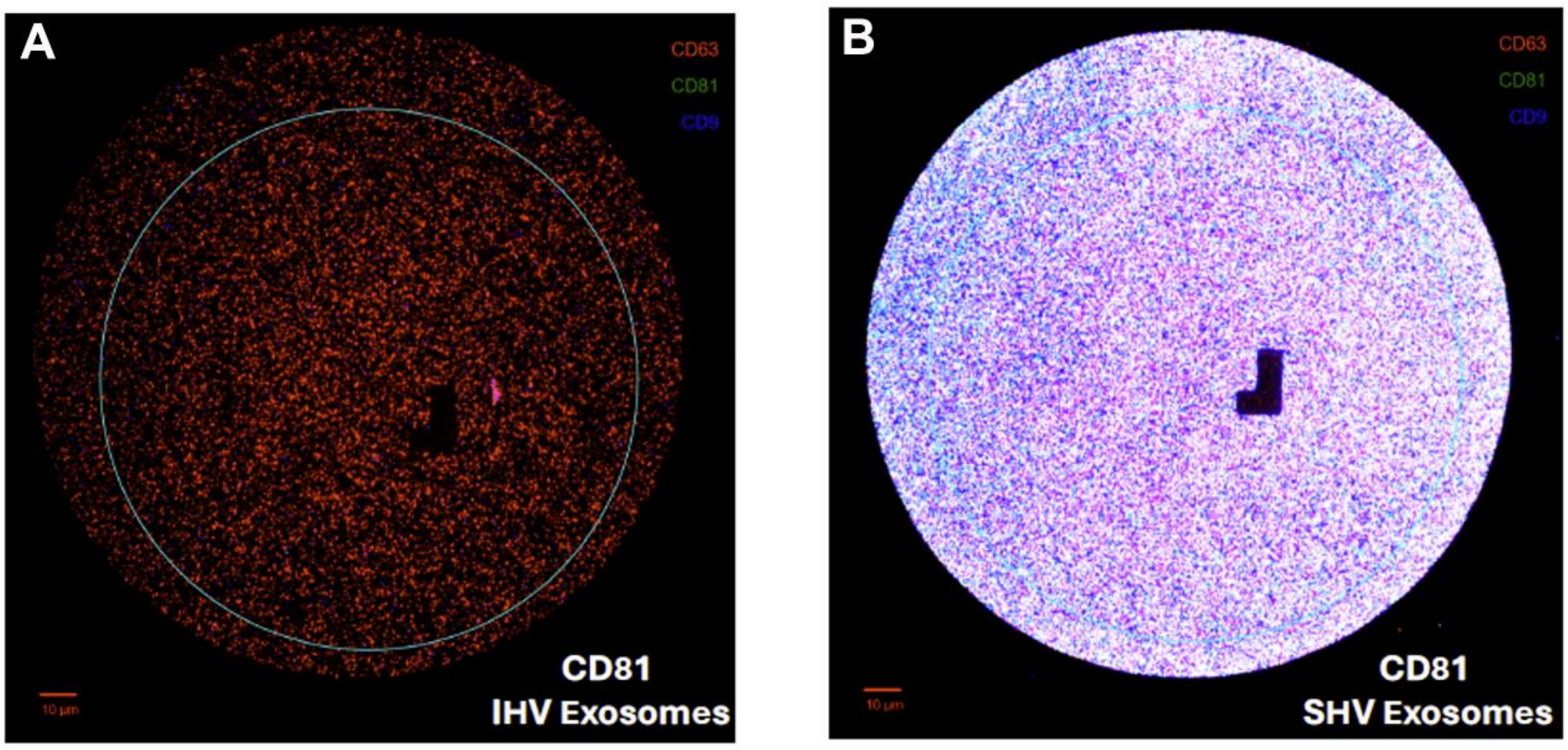

Supplementary Figure 5. CD81 exosomes are elevated in SHEV compared with IHEV as determined by Leprechaun analysis. (A) Example of CD81-captured exosomes from IHEV co-stained for the tetraspanins CD63, CD81, and CD9; (B) CD81-captured exosomes from SHEV stained in the same manner. EVs were from the same human heart. SHEV has more CD81 exosomes and more that co-express other tetraspanins. SHEV: Subsarcolemmal heart extracellular vesicle; IHEV: interfibrillar heart extracellular vesicle; EV: extracellular vesicle.

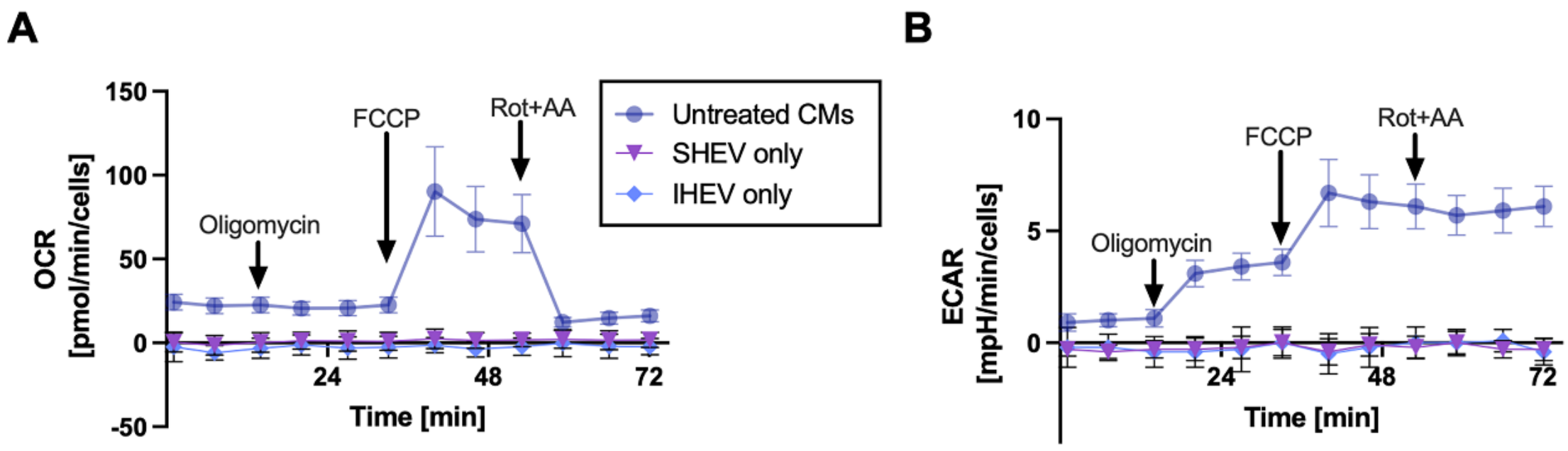

Supplementary Figure 6. SHEV and IHEV preparations exhibit no detectable intrinsic respiratory activity. (A) OCR and (B) ECAR were measured in Seahorse wells containing SHEV or IHEV preparations in the absence of NRVMs and compared with untreated cardiomyocytes (CMs). SHEV and IHEV preparations alone exhibited no detectable oxygen consumption or extracellular acidification under these assay conditions, indicating that the EV preparations did not contribute measurable intrinsic respiratory activity to the responses observed in EV-treated NRVMs. Data represent technical Seahorse wells; error bars indicate SEM. Arrows indicate substrate or inhibitor additions. SHEV: Subsarcolemmal heart extracellular vesicle; IHEV: interfibrillar heart extracellular vesicle; OCR: oxygen consumption rate; ECAR:

extracellular acidification rate; NRVM: neonatal rat ventricular myocyte; CM: cardiomyocyte; EV: extracellular vesicle; SEM: standard error of the mean.

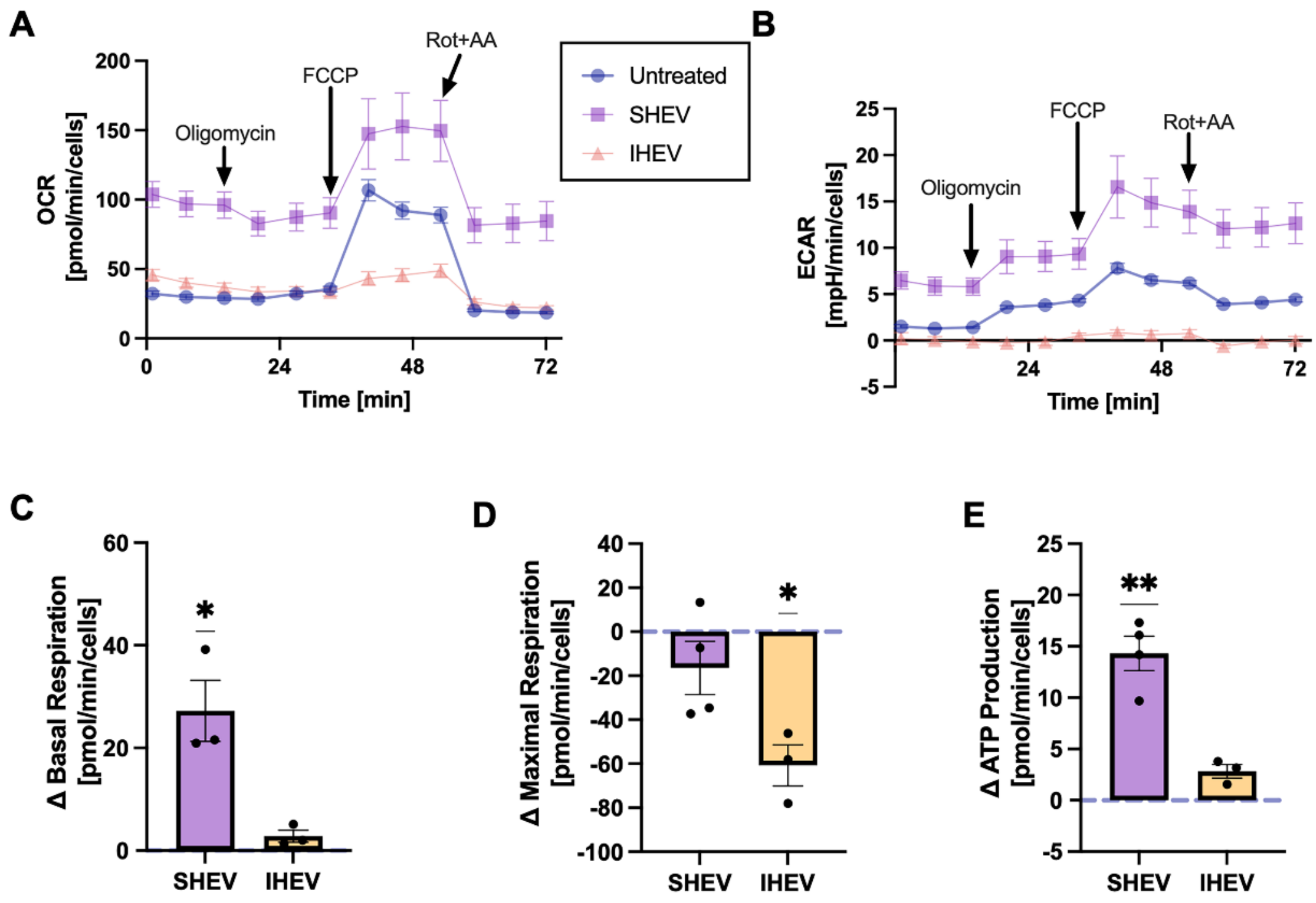

Supplementary Figure 7. Independent replication of SHEV- and IHEV-associated effects on cardiomyocyte bioenergetics. An independent NRVM preparation was treated for 48 h with the same donor-derived SHEV and IHEV preparations used in the particle-normalized experiment shown in Figure 9. In this experiment, equal volumes (10 µL/well) of each EV preparation were administered. Retrospective calculation using NTA-derived particle concentrations indicated EV exposures ranging from approximately 1.82×10^8 to 8.40×10^8 particles/well. (A) OCR and (B) ECAR in untreated, SHEV-treated, and IHEV-treated NRVMs. Arrows indicate substrate or inhibitor additions. (C) Change in basal respiration, (D) maximal respiration, and (E) ATP production relative to the mean untreated response from the same NRVM preparation (Δ = EV-treated donor mean − untreated mean). Each point in C–E represents one independently derived human EV donor preparation and is the mean of technical Seahorse wells. The dashed horizontal line at Δ=0 represents the untreated NRVM reference. Donor-level Δ values were compared with zero using two-sided one-sample t-tests. *$p<0.05$; **$p<0.01$. Error bars indicate SEM. NRVM: Neonatal rat ventricular myocyte; SHEV: subsarcolemmal heart extracellular vesicle; IHEV: interfibrillar heart extracellular vesicle; EV: extracellular vesicle; NTA:

nanoparticle tracking analysis; OCR: oxygen consumption rate; ECAR: extracellular acidification rate; ATP: adenosine triphosphate; SEM: standard error of the mean.

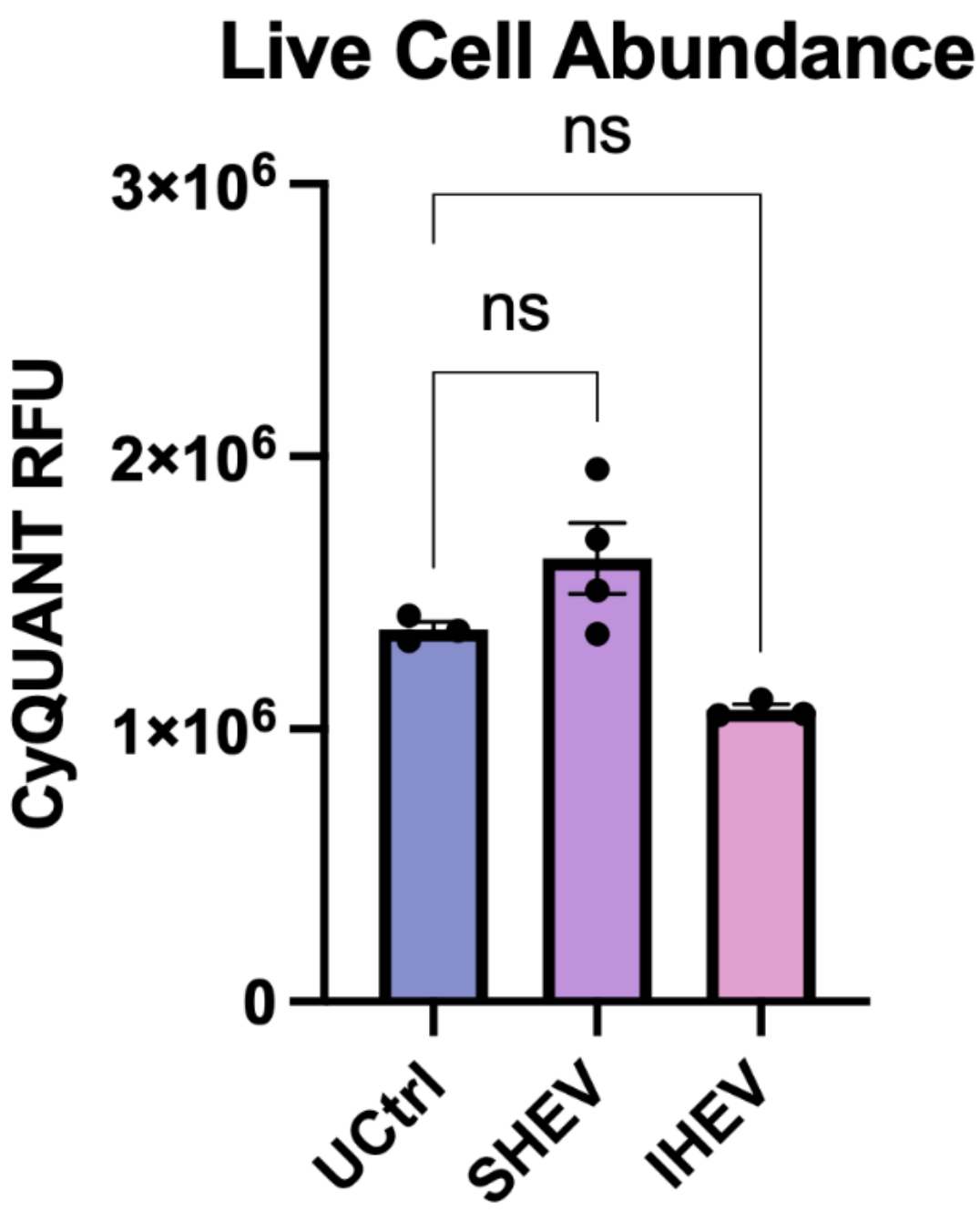

Supplementary Figure 8. CyQUANT assessment of viable-cell abundance following EV treatment. Viability of SHEV- or IHEV-treated cardiomyocytes compared with untreated control cardiomyocytes (Uctrl). Analysis was performed using ordinary one-way ANOVA with multiple comparisons to untreated cells corrected using Tukey's test. Each data point for SHEV and IHEV treated cells is the average of five separate wells per condition. Data points for untreated cells represent individual Seahorse wells. Error bars show SEM. EV: Extracellular vesicle; SHEV: subsarcolemmal heart extracellular vesicle; IHEV: interfibrillar heart extracellular vesicle; Uctrl: untreated control; ANOVA: analysis of variance; SEM: standard error of the mean.

Supplementary Table 1. Number of side chain carbons and double bonds in phospholipid species analyzed for this study

CARDIOLIPIN			PHOSPHATIDIC ACID			PHOSPHATIDYLGLYCEROL		
**Mass/Charge**	**Carbons**	**Double Bonds**	**Mass/Charge**	**Carbons**	**Double Bonds**	**Mass/Charge**	**Carbons**	**Double Bonds**
**CL 1394**	68	7	**PA 671**	34	2	**PG 745**	34	2
**CL 1396**	68	6	**PA 673**	34	1	**PG 747**	34	1
**CL 1398**	68	5	**PA 675**	34	0	**PG 773**	36	2
**CL 1422**	70	5	**PA 695**	36	4	**PG 775**	36	1
**CL 1424**	70	6	**PA 697**	36	3			
**CL 1448**	72	8	**PA 699**	36	2	**PHOSPHATIDYLINOSITOL**		
**CL 1450**	72	7	**PA 701**	36	1	**PI 857**	36	4
**CL 1452**	72	6				**PI 859**	36	3
**CL 1454**	72	5	**PHOSPHATIDYLCHOLINE**			**PI 861**	36	2
**CL 1456**	72	4	**PC 790**	32	1	**PI 885**	38	4
**CL 1470**	74	11	**PC 792**	32	0	**PI 887**	38	3
**CL 1472**	74	10	**PC 816**	34	2	**PI 889**	38	2
**CL 1474**	74	9	**PC 818**	34	1	**PI 891**	38	1
**CL 1476**	74	8	**PC 840**	36	4	**PI 909**	40	6
**CL 1494**	76	13	**PC 842**	36	3	**PI 911**	40	5
**CL 1496**	76	12	**PC 844**	36	2	**PI 913**	40	4

**CL 1498**	76	11	**PC 864**	38	6			
**CL 1518**	78	15	**PC 866**	38	5	**PHOSPHATIDYLSERINE**		
**CL 1520**	78	14				**PS 758**	34	2
**CL 1522**	78	13	**PHOSPHATIDYLETHANOLAMINE**			**PS 760**	34	1
**CL 1524**	78	12	**PE 714**	34	2	**PS 762**	34	0
**CL 1544**	80	16	**PE 716**	34	1	**PS 786**	36	2
**CL 1546**	80	15	**PE 738**	36	4	**PS 788**	36	1
**CL 1548**	80	14	**PE 740**	36	3	**PS 810**	38	4
			**PE 742**	36	2	**PS 834**	40	6
**MONOLYSOCARDIOLIPIN**			**PE 762**	38	5			
**mlcl 1160**	52	5	**PE 764**	38	4	**SPHINGOMYELIN**		
**mlcl 1162**	52	4	**PE 766**	38	3	**SM 789**	18	0
**mlcl 1186**	54	6	**PE 788**	40	5	**SM 817**	20	0
**mlcl 1188**	54	5	**PE 790**	40	6	**SM 843**	22	1
**mlcl 1210**	56	8				**SM 845**	22	0
**mlcl 1212**	56	7				**SM 869**	24	2
						**SM 871**	24	1

CL: Cardiolipin; PA: phosphatidic acid; PG: phosphatidylglycerol; PC: phosphatidylcholine; PI: phosphatidylinositol; PE: phosphatidylethanolamine; PS: phosphatidylserine; SM: sphingomyelin; mlcl: monolysocardiolipin.

Supplementary Table 2. Statistics for protein function bar graphs in Figure 5 using ordinary one-way ANOVA corrected for multiple comparisons using Tukey's test

**Tukey's multiple comparisons test**	**Mitochondrial & glycolysis**	**Sarcomere cytoskeleton**	**Vesicle trafficking**	**ER, Golgi & secretory**	**Other non-mitochondrial**
SSL Mito *vs*. IF Mito	**0.0032**	**0.0372**	**0.027**	0.9797	0.9512
SSL Mito *vs*. SHEV	**< 0.0001**	**< 0.0001**	**0.0016**	**0.027**	0.1614
SSL Mito *vs*. IHEV	**< 0.0001**	**< 0.0001**	**< 0.0001**	0.0777	**0.0039**
SSL Mito *vs*. Heart	**< 0.0001**	**< 0.0001**	**0.0001**	**< 0.0001**	0.057
IF Mito *vs*. SHEV	**< 0.0001**	**< 0.0001**	0.5676	**0.011**	0.05
IF Mito *vs*. IHEV	**< 0.0001**	**< 0.0001**	**0.0015**	**0.0309**	**0.0117**
IF Mito *vs*. Heart	**< 0.0001**	**< 0.0001**	**0.0092**	**< 0.0001**	**0.0206**
SHEV *vs*. IHEV	0.1663	**0.0043**	**0.0029**	0.8946	**< 0.0001**
SHEV *vs*. Heart	**0.0044**	**< 0.0001**	**0.0296**	**< 0.0001**	0.7384
IHEV *vs*. Heart	**0.0004**	**< 0.0001**	0.9527	**< 0.0001**	**< 0.0001**

SSL: Subsarcolemmal; IF: interfibrillar; Mito: mitochondria; SHEV: subsarcolemmal heart extracellular vesicle; IHEV: interfibrillar heart extracellular vesicle; ANOVA: analysis of variance.
